# Eight Nucleotide Substitutions Inhibit Splicing to HPV-16 3′-Splice Site SA3358 and Reduce the Efficiency by which HPV-16 Increases the Life Span of Primary Human Keratinocytes

**DOI:** 10.1371/journal.pone.0072776

**Published:** 2013-09-09

**Authors:** Xiaoze Li, Cecilia Johansson, Carlos Cardoso Palacios, Anki Mossberg, Soniya Dhanjal, Monika Bergvall, Stefan Schwartz

**Affiliations:** Department of Laboratory Medicine, Lund University, Lund, Sweden; National Institute of Health – National Cancer Institute, United States of America

## Abstract

The most commonly used 3′-splice site on the human papillomavirus type 16 (HPV-16) genome named SA3358 is used to produce HPV-16 early mRNAs encoding E4, E5, E6 and E7, and late mRNAs encoding L1 and L2. We have previously shown that SA3358 is suboptimal and is totally dependent on a downstream splicing enhancer containingmultiple potential ASF/SF2 binding sites. Here weshow that only one of the predicted ASF/SF2 sites accounts for the majority of the enhancer activity. We demonstrate that single nucleotide substitutions in this predicted ASF/SF2 site impair enhancer function and that this correlates with less efficient binding to ASF/SF2 in vitro. We provide evidence that HPV-16 mRNAs that arespliced to SA3358 interact with ASF/SF2 in living cells. In addition,mutational inactivation of the ASF/SF2 site weakened the enhancer at SA3358 in episomal forms of the HPV-16 genome, indicating that the enhancer is active in the context of the full HPV-16 genome.This resulted in induction of HPV-16 late gene expression as a result of competition from late splice site SA5639. Furthermore, inactivation of the ASF/SF2 site of the SA3358 splicing enhancer reduced the ability of E6- and E7-encoding HPV-16 plasmids to increase the life span of primary keratinocytes in vitro, demonstrating arequirement for an intact splicing enhancer of SA3358 forefficient production of the E6 and E7 mRNAs. These results link the strength of the HPV-16 SA3358 splicing enhancer to expression of E6 and E7 and to the pathogenic properties of HPV-16.

## Introduction

A long-term persistent infection with one of the high-risk human papillomaviruses (HPVs) is the highest risk factor for development of cervical cancer [1], [2]. HPVs are present in virtually all cases of cervical cancer and HPV-16 is by far the most common of the cancer-associated HPV types [3]. Although HPV-16 infections may persist and cause cancer, the majority of these are productive infections that are cleared by the host [4]. Why some HPV-infections persist to cause high-grade cervical lesions and cancer is currently unknown, although host factors are clearly contributing. Persistent HPV-16 infections that progress to cancer are characterised by dysregulated viral gene expression, i.e continuous E6 and E7 expression, and lack of late L1 and L2 expression. Since this dysregulation may contribute to cancer progression, it is important to understand how HPV-16 gene expression is regulated.

**Figure 1:**
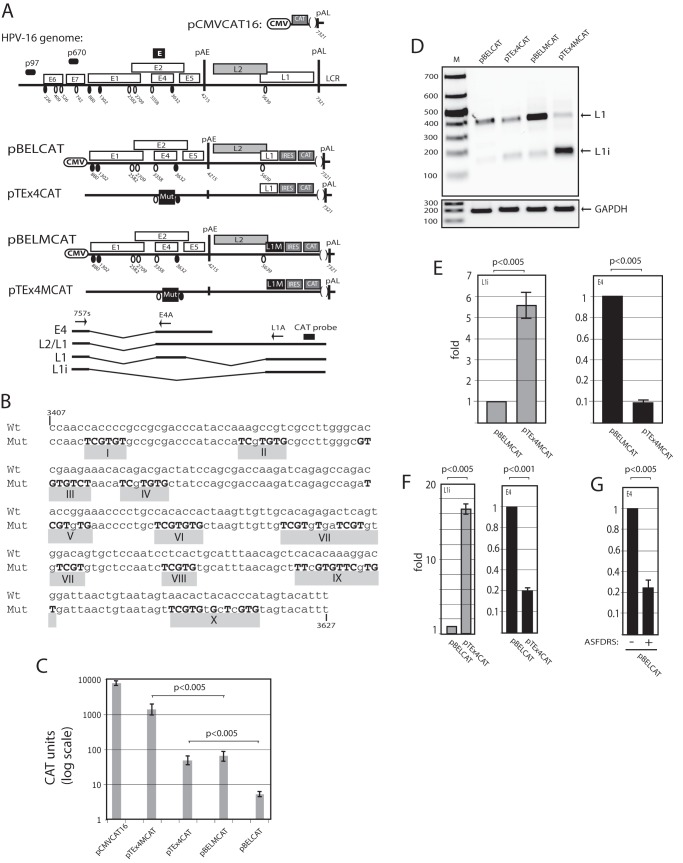
Characterisation of the splicing enhancer downstream of HPV-16 3′-splice site SA3358 using novel HPV-16 reporter plasmids. (**A**). Schematic representation of the HPV-16 genome, the subgenomic HPV-16 expression plasmids and the control plasmid pCMVCAT16 [39]. The early and late viral promoters p97 and p670 are indicated. Numbers indicate nucleotide positions of 5′- (filled circles) and 3′-splice sites (open circles) or the early and late poly (A) sites pAE and pAL, respectively, and the borders of deletions. L1M represents a previously described mutant HPV-16 L1 sequence in which a number of nucleotide substitutions that inactivate splicing silencers have been inserted downstream of SA5639. IRES, the poliovirus internal ribosome entry site sequence; CAT, CAT reporter gene; CMV, human cytomegalovirus immediate early promoter; LCR, long control region. mRNAs produced by the plasmids are indicated. The position of the CAT probe and RT-PCR primers (arrows) are indicated. (**B**) The HPV-16 sequence between nucleotides 3407 and 3627 in the exon between SA3358 and SD3632 is displayed.The previously identified 10 clusters of 15 ASF/SF2 bind sites [18] predicted by ESE finder [43]. (**C**) CAT protein levels produced in transfected cells. CAT was monitored as described in Materials and Methods. Mean values and standard deviations are shown. Note the logarithmic scale. (**D**) RT-PCR with primers 757s and L1A on cDNA of cytoplasmic RNA extracted from HeLa cells transfected with the indicated plasmids.L1 and L1i mRNAs are indicated. The RT-PCR is qualitative only, as it has not been optimised for RNA quantitation. M, molecular weight marker; GAPDH,cDNA amplified as internal control. (**E, F**) Real time PCR of HPV-16 E4 mRNAs using primers 757s and E4A and L1i mRNAs with primers 757s and L1A from as described in Materials and Methods. (**G**) Real time PCR of HPV-16 E4 mRNAsproduced from pBELCAT in the absence of presence of cotransfectedpASFDRS plasmid. RT-PCR primers were 757s and E4A.

Transcription of HPV-16 mRNAs initially occurs from the early p97 promoter but latershifts to the cell-differentiation dependent, late promoter p670 (Fig 1A) [5], [6], [7], [8], [9]. Alternative polyadenylation and splicing is required for ordered expression of the viral genes, and viral splice sites and polyA signals are regulated by viral and cellular factors [10], [11], [12], [13], [14]. For example, the HPV-16 E2 protein induces HPV-16 late gene expression by inhibiting the early HPV-16 polyA signal pAE [13]. This polyA signal is also under control of cellular factors such as CPSF-30 [13], hnRNP H [15], CstF-64 [15], [16] and HuR [13]. The major HPV-16 3′-splice site SA3358 (Fig 1A) is under control of ASF/SF2, SRp30c and SRp20 [17], [18], [19], and is likely to be required for production of HPV-16 mRNAs encoding E4, E5, E6, E7, L1 and L2, whereas E1 and E2 expression is negatively affected by efficient usage of SA3358 [20]. Other HPV types have splice sites that correspond in function and location to HPV-16 SA3358 and SD880, i.e. they are typically used to generate the E1-E4 fusion protein by splicing. These sites are named SD847 and SA3325 in HPV-11 and SD877 and SA3295 in HPV-31 [20]. The most common HPV-16 E6 and E7 mRNAs are spliced between HPV-16 SD880 and SA3358 [21], [22], [23], or the corresponding sites in HPV-11 [24], [25], [26] and HPV-31 [27], [28]. In addition, the most-common late mRNAsencoding E4, L1 and L2 are also spliced between HPV-16 SD880 and SA3358 [29], or the corresponding sites in HPV-11 [26] and HPV-31 [28], [30]. In vivo, HPV-16 splicing between SD880 and SA3358 was the most-common splicing event in both low- and high-grade cervical lesions [31], suggesting that SA3358 not only plays an important role during a productive HPV-16 infection, it is also likely to be important for pathogenesis. It is reasonable to speculate that SA3358 is highly regulated during the HPV-16 life cycle, and that factors regulating it may be involved in the aberrant HPV-16 gene expression profile that is observed in high grade genital lesions and cervical cancer. In conclusion, the efficient usage of HPV-16 3′-splice site SA3358 in the life cycle and pathogenesis of HPV-16 as well as its conservation in other HPV types, suggest that a better understanding of the regulation of HPV-16 SA3358 will enhance our understanding of the life cycle and pathogenesis of the papillomaviruses.

We have previously shown that SA3358 is suboptimal due to the lack of an uninterrupted polypyrimidine tract immediately upstream of the invariable AG dinucleotide [32], and that SA3358 therefore is dependent on a downstream splicing enhancer [32]. Subsequent experiments from our group revealed that that the exon between SA3358 and SD3632 contains 10 clusters of 15 ASF/SF2 binding sites predicted by ESEfinder [18]. Each cluster consisted of 1–3 overlapping ASF/SF2 sites. Mutational inactivation of all predicted ASF/SF2 sites destroyed the enhancer and caused skipping of SA3358, which resulted in alternative splicing to the downstream late, L1 3′-splice site SA5639 [18]. Jia et al also identified a sequence located downstream of HPV-16 SA3358 that enhanced splicing to SA3358. Binding of SRp20 to this sequence inhibited splicing to SA3358 [19]. Since the activity of SA3358 must change during the viral life cycle, one may assume that it is under control of multiple cellular factors, in particular various SR proteins as their functions often overlap [33]. Overexpression of SRp30cinhibited SA3358 and caused skipping of the exon between SA3358 and SD3632, which resulted in splicing to late L1 splice site SA5639 instead of SA3358 [17]. However, ASF/SF2 is of particular interest since it has been defined as a proto-oncogene [34] and since it ishighly expressed in cervical cancers [35], [36].One may therefore speculate that it can enhance the oncogenic properties of HPV-16.

This is a follow up of our previous studies of the SA3358 splicing enhancer [18], [32] in which we now demonstrate that only one of the 15 predicted ASF/SF2 sites accounts for the majority of the enhancer activity, while the others contribute minimally. We identify single nucleotide substitutions in this predicted ASF/SF2 site that severely incapacitate the enhancer and we show that the enhancer is active in primary human epithelial cells.In addition, mutational inactivation of the enhancer, inducedlate gene expression from episomal forms of the complete HPV-16 genome, both in cervical cancer cells and in primary human keratinocytes. We also show that HPV-16 mRNAs which are spliced to SA3358, interact with ASF/SF2 in living cells and that mutations that reduce the enhancer activity of the ASF/SF2 site, also interact less well with ASF/SF2. Furthermore, inactivation of the ASF/SF2 site of the splicing enhancer reduced the ability of subgenomic HPV-16 plasmids to increase the life span of primary keratinocytes in vitro, demonstrating the importance of SA3358 for the expression of E6 and E7 mRNAs.These experiments link the activity of the HPV-16 splicing enhancer at SA3358 to the pathogenic properties of HPV-16.

## Materials and Methods

### Plasmid construction

The following plasmids have been described previously: pCAGGS-nlscre [37], pTEx4 [18], pTEx4M [18],pBELMCAT [38], pBELCAT [38], pASFDRS [18]and pCMVCAT16 [39].To generate pTEx4CAT and pTEx4MCAT, an XhoI-MluI fragment was excised from pBELMCAT [38] and inserted into pTEx4 and pTEx4M, respectively. To generate pBELMH1, pBELMH4, pBELMH5, pBELMH6, pBELMH18 and pBELMH41, PCR was first carried out on pHPV1, pHPV4, pHPV5, pHPV6, pHPV18 and pHPV41 with oligonucleotides 1MS and 1MAS, 4S and 4AS, 5S and 5AS, 6S and 6AS, 18S and 18AS, 41S and 41AS, respectively. The amplified fragments were digested with BssHII and XbaI and subcloned into pBELMCAT [38], resulting in pBELMH1, pBELMH4, pBELMH5, pBELMH6, pBELMH18 and pBELMH41, respectively.To generate pI, pI+II, pI+II+III, pI+II+III+IV, pI+II+III+IV+V, pI+II+III+IV+V+VI, pIII, pIII+IV, pV+VI, pVII+VIII and pVIII site-directed mutagenesis was performed on pBELMCAT [38] by using a QuikChangeII XL Site-Directed Mutagenesis kit (Stratagene) with forward and reverse primer pairs.Mutants were made in an accumulative mannerand plasmids were sequenced after each mutagenesis step. Mutant plasmidspVII, pIX and pX were generated by subcloninginto pBELMCAT [38] of BssHII-XbaI mutant HPV-16 gene segments ordered from Eurofins MWG Operon. To generate pC97ELMCAT, pBearly97 [40] was digested with SalI and BssHII and the excised fragment was subcloned into pBELMCAT [38], resulting in pC97ELMCAT. To generate pC97-III*MCAT, pIIIwere digested with XbaI and BssHII and subcloned into pC97ELMCAT, resulting in pC97-III*MCAT.

To generate pBELluc and pBELMluc, the luciferase gene was PCR amplified with lucS and lucA. The PCR fragment was digested with MluI and XhoI and subcloned into pBELCAT [38] and pBELMCAT [38]. A BamHI-XhoIfragment was released from pBELMlucand subcloned into pIII, generating p3*luc. To generate p3*1ME2luc, p3*2ME2luc, p3*3ME2luc, p3*4ME2luc, p3*5ME2luc, p3*6ME2luc and p3*ME4luc, site-directed, ligase-independent mutagenesis (SLIM) was performed on pIII*luc by using oligonucleotides 135FT and 136RT, 137FT and 138RT, 156FT and 157RT, 139FT and 140RT, 160FT and 161RT, 162FT and 163RT, 143FT and 164RT, respectively, and the same 130RS, 128FS oligonucleotides in all reactions. Primer sequences are available on request.

The secreted luciferase (sluc) coding sequence was PCR-amplified by primers slucS and sLucA and subcloned into pCR2.1-TOPO (Invitrogen). The sluc gene was released with MluI and XhoI and subcloned into p3*luc, pBELCAT [38]orpBELMluc, generating p3*sluc, pBELsluc and pBELMsluc. To generate p3*1ME2sluc, p3*2ME2sluc and p3*ME4sluc,MluI and XhoI digested sluc gene was inserted into p3*1ME2luc, p3*2ME2luc and p3*ME4luc, thereby replacing the luciferase gene with sluc. To generate pC97ELsluc and pC97EL-III*sluc, pBELsluc was digested with ApaI and XhoI and subcloned intopC97ELMCAT and pC97-III*MCAT, generating pC97ELsluc and pC97EL-III*sluc. To generate pC97EL-1ME2sL and pC97EL-4ME2sL, p3*1ME2luc and p3*4ME2luc were digested with XbaI and BssHII and the excised fragments were subcloned into pC97ELsluc, resulting in pC97EL-1ME2sL and pC97EL-4ME2sL.

### HPV-16 genomic plasmids

The complete HPV-16 genome was PCR-amplified and inserted into TOPO followed by regeneration of ends upto the unique SphI site. A 34-nucleotide LoxP site was inserted immediately adjacent to the SphI sites flanking the HPV-16 genome. AnSphI fragment was deleted from the TOPO vector to generate a plasmid containing only two SphI sites. A sequence encoding the Rous sarcoma virus long terminal repeat followed by the neomycin resistance gene and the simian virus 40 polyA signal was synthesized by Eurofins MWG GmbH. This sequence was inserted downstream of the LoxP site in the genomic HPV-16 plasmid, resulting in pHPV16AN. Introduction of an XhoI site downstream of the L1 stop codon, and insertion of the poliovirus type 2A internal ribosome entry site followed by the gene encoding the secreted luciferase between the unique BamHI- and XhoI-sites, resulted in the pHPV16ANSL plasmid. pHPV16MANSL was generated by insertion of a ApaI-BamHI fragment from pBELM into pHPV16ANSL. To generate mutant HPV-16 genomes, a sequence from SexAI to ApaI in the HPV-16 genome was PCR amplified and inserted into pCRTOPO, generating pEI. Site-directed, ligase-independent mutagenesis (SLIM) was performed on pEI by using oligonucleotides 127FT and 129RT, 135FT and 136RT, 158FT and 159RT, 139FT and 140RT, respectively, and the same 130RS, 128FS oligonucleotides in all reactions, generating pTOPIII*, pTOPE1ME2, pTOPE4ME2, pTOPIII*ME4. To generate pHPV16ANSL-III*, pHPV16MANSL-III*, pHPV16MANSL-1ME2, pHPV16MANSL-4ME2 and pHPV16MANSL-III*ME4, a fragment was released with SexAIand ApaIfrom pTOPIII*,pTOPE1ME2, pTOPE4ME2 or pTOPIII*ME4 and subcloned into pHPV16ANSL or pHPV16MANSL, generating pHPV16ANsL-III*, pHPV16MANSL-III*, pHPV16MASL-1ME2, pHPV16MANSL-4ME2, pHPV16MANSL-III*ME4. pRSVneo was synthesised by Eurofins MWG Operon and contains the Rous sarcoma virus long terminal repeats promoter followed by the neomycine resistance gene and a simian virus 40 late polyA signal. The origin and properties of the plasmids used here are summarised in Table S1. Primers are listed in Table S2.

### Transfection of HeLa, C33A and 293T cells

HeLa cells were cultured in Dulbecco's modified Eagle medium with 10% heat-inactivated foetal bovine calf serum and penicillin-streptomycin. Transfections were carried out using Turbofect according to the manufacturer's instructions (Fermentas). Briefly, the mixture of 2 ul of Turbofect and 100 ul of Dulbecco's Modified Eagle Medium without serum was added to 1 ug of plasmid DNA and incubated at room temperature for 15 min prior to drop-wise addition to 60 mm plates with subconfluentHeLa cells. Plasmids pCMVSEAP or pCMVsLuc are included in the transfection experiments to control for transfection efficiency.Cells were harvested at 24 h posttransfection. Each plasmid was transfected in triplicate, in a minimum of two independent experiments.

### Propagation and transfection of human primary keratinocytes

Neonatal human epidermal keratinocytes (HEKn) were purchased from Gibco Invitrogen Cell Culture, and were propagated in EpiLife Medium supplemented with Human keratinocyte Growth Supplement (Gibco Invitrogen Cell Culture) as recommended by the manufacturer. The primary keratinocytes are grown in monolayer cultures and expanded under growth conditions that maintain poorly differentiated cells with properties similar to the cells in the basal layers of the epithelium.Cells passaged less than three times were used. 150 000 cells were seeded per 60 mm plate for each transfection. Co-transfections of genomic HPV-16 plasmids and plasmid pCAGGS-nlscre [37] (generously provided by Dr.Andras Nagy at University of Toronto), which expresses the crerecombinase, were carried out using Fugene 6 (Roche), as previously described for HPV-18 [41]. Briefly, the mixture of 9ul of Fugene 6 and 200 ul of EpiLife Medium with supplements was added to 3 ug of plasmid DNA and incubated at room temperature for 15 min prior to dropwise addition to 60 mm plates with subconfluentHEKn cells. At 24 hrs after transfection, G418 was added to the cells at a concentration of 100 ug/ml. Medium was harvested for analysis of secreted luciferase at different time points after transfection. Total RNA or DNA analysis was performed at day 6 post transfection. Each plasmid was transfected in triplicates, in a minimum of two independent experiments.

### Assay for extended life-time of primary keratinocytes

Neonatal humanepidermal keratinocytes (HEKn) were transfected as described above. Co-transfections of pC97ELsluc or pC97ELslucp3* with pRSVneowere carried out using Fugene 6 (Roche), as previously described for HPV-18 [41].At 24 hrs posttransfection, G418 was added to the cells every second day for four days at a concentration of 100 ug/ml. After these four days, medium without G418 was changed once a week. Approximately 45 days after transfection when cell colonies had grown to 2–5 mm of diameter, they were picked and transferred to 12-well plates. Each plasmid was transfected in triplicates in multiple independent experiments.

### CAT ELISA

Cell extracts were prepared from transfected HeLa cells at 24 h posttransfection according to the protocol from a CAT ELISA kit (Roche). All cell extracts were appropriately diluted to obtain an absorbance reading in the linear range of the CAT ELISA assay. To be able to compare CAT results between experiments, pCMVCAT16 was transfected in triplicate in each transfection experiment. Arbitrary CAT units were calculated by multiplying the absorbance 405 readings with the dilution factor for each sample. Mean values and standard deviations were calculated for all samples.

### Luciferase assay

HeLa cells transfected in 60 mm plateswere lysed in 150ullysisbuffer (25 mM Tris, pH7.8, 2 mM DTT, 10% glycerol 1% Triton X-100) after 24 h post-transfection.Luciferase activity was monitoredby mixing in 20 ul cell lysate and 30 ul luciferase substrate (20 mM Tricine pH7.8, 1.07 mM MgCO_3_, 2.67 mM MgSO_4_, 0.1 mM EDTA, 33.3 mM dithiothreitol (DTT), 270 uM coenzyme A, 470 uM luciferine and 530 um ATP). Luminescence was monitored using a Tristar LB941 Luminometer.

### Secreted luciferase assay

The *Metridia longa* secreted luciferase activity in the medium of the transfected cells was monitored with the help of the Ready To Glow Secreted Luciferase Reporter assay according the instructions of the manufacturer (Clontech). Briefly, 50 ul of cell culture medium was added to 5 ul of 0.5X Secreted Luciferase substrate/Reaction buffer in a 96-well plate and luminiscence was monitored in a Tristar LB941 Luminometer.

### DNA extraction

Cellular DNA was extracted after lysis of cells in lysis buffer (10 mM Tris - HCl, pH 8.0-buffer supplemented with 100 mM NaCl, 25 mM EDTA, 0.5% SDS and 250 ug proteinase K). The cell extracts were incubated at 50°C over. An equal volume of phenol/chloroform/isoamyl alcohol (chisam) was added to each cell extract. The aqueous phase was collected after centrifugation of the samples, and subjected to ethanol precipitation. The samples were treated with RNaseA and stored at −20°C until use. To monitor recombination at the loxP sites in pHPV16AN and pHPV16ANSL, PCR was performed with primers 16S (5′-TATGTATGGTATAATAAACACGTGTGTATGTG-3′) and 16A (5′-GCAGTGCAGGTCAGGAAAACAGGGATTTGGC-3′). (This PCR reaction yields a 366-nucleotide PCR fragment that is diagnostic for recombination at the LoxP sites.

### RNA extraction, reverse transcription (RT)-PCR andqPCR

Cytoplasmic RNA was extracted 24h posttransfection using NP-40 buffer as described previously. Total RNA was extracted by Tri reagent (Sigma) according to manufacturers protocol. Two hundred nanograms of cytoplasmic RNA were reverse transcribed in a 25ul reaction at 42°C by using Superscript II and random hexamers according to the protocol of the manufacturer (Invitrogen). Two microliter of cDNAwere subjected for PCR amplification of cDNA. Real time PCR was performed in a MiniOpticon (BioRad) using the Sso Advanced SYBR Green Supermix (BioRad) according the manufacturers' instructions. HPV-16 E6*I/E7 mRNAs spliced from SD226 to SA409 were amplified with primers E6S and 757as, E2 mRNAs spliced from SD880 to SA2709 were amplified with primers 757S and ExonE2as, E4 mRNAs spliced from SD880 to SA3359 were amplified with primers 757S and E4A (Fig 1A) and L1 and L1i mRNAs were amplified with primers 757S and L1A (Fig 1A) (Primers are listed in Table S2). The two bands representing L1 and L1i were separated with the help of melt curves and could be separately quantified. The results were normalised to GAPDH mRNA levels.

### Intracellular UV cross-linking and immunoprecipitation of RNA-protein complexes

Transfected 293T cells in 10 cm dishes were washed with ice-cold PBS and UV-irradiated at 400 mJ/cm^2^ in a Bio-link cross-linker (Biometra). Cells were lysed in 500 ul of lysis buffer (0.3 M NaCl, 50 mM Tris, pH7.4, 0.5% NP-40, protease inhibitors, Riboblock (Fermentas)). Cells were incubated on ice for 30 min with occasional vortexing to lyse cells. For immunoprecipitations, anti-ASF/SF2 monoclonal antibody clone 96 (Invitrogen) or normal mosueIgG sc-2025 (Santa Cruz Biotechnlogy, Inc)were incubated with the cleared cell lysates for overnight at 4°C. Protein G magnetic beads were added and incubation continued for another one hour at 4°C. The beads were washed 5x with lysis buffer and RNA was eluted by phenol/chloroform-extracted and ethanol precipitated. Nine microliter of immunoprecipitated RNA were reverse transcribed in a 25 ul reaction at 42°C by using Superscript II and random hexamers according to the protocol of the manufacturer (Invitrogen). Two microliter of cDNAwere subjected to PCR amplification using oligonucleotides indicated in the Figure 1A.

### RNA-protein pull-down assay and Western blotting

Nuclear extracts were prepared from subconfluentHeLa cells according to the procedure described by Dignam et al [42]. HeLa nuclear extract was mixed with streptavidin coated magnetic beads (Invitrogen) bound to biotin-labeled RNA oligonucleotides in 500 ul of binding buffer (10 mM Tris, pH7.4, 200 mM NaCl, 2.5 mM MgCl_2_, 0.5% Triton X-100). After incubation with rotation for 1 h at 4°C, beads were washed 5 times in binding buffer. Proteins were eluted by boiling the beads in SDS-PAGE loading buffer and subjected to Western blotting.

Western blotting was performed by separating proteins on SDS-PAGE gels followed by transfer to nylon membranes (Whatman) andWestern blot analysis with anti-ASF/SF2 monoclonal antibody clone 96 (Invitrogen), anti-SF2 antibody (ab38017, Abcam), anti-Rb antibody (ab6075, Abcam) oranti-p53 antibody (ab131442, Abcam).

## Results

### Characterisation of the splicing enhancer downstream of HPV-16 3′-splice site SA3358 using novel HPV-16 reporter plasmids

We have previously identified a splicing enhancer (E) downstream of HPV-16 3′-splice site SA3358 [18], [32], the most commonly used splice site in the HPV-16 genome (Fig 1A). With the help of ESE finder [43] we predicted 10 clusters of 15 potential ASF/SF2 binding sites (Fig 1B) [18].Each cluster consisted of one or more overlapping predicted ASF/SF2 binding sitelocated betweennucleotide positions 3407 and 3627 in the HPV-16 genome (Fig 1B) [18]. We have previously shown that mutational inactivation ofall ten clusters destroyed the enhancer activity [18]. Here we have characterised this enhancer further by investigating the contribution of each predicted ASF/SF2 cluster to the enhancer activity. To perform these studies we used a recently described reporter assay that allowed us to quantitate the contribution of each cluster to the enhancer activity [38]. It is based on an HPV-16 reporter plasmid named pBEL [44]in which the last two thirds of the L1 gene had been replaced by the poliovirus 2A internal ribosome entry site followed by the CAT reporter gene, resulting in the pBELCATreporter plasmid (Fig 1A) [38].This plasmid producedlow levels of CAT upon transfection of HeLa cells as the majority of the mRNAs are spliced to SA3358 and polyadeylated at the early polyA signal pAE, thereby primarily generating E4 mRNAs that do not encode CAT (Fig 1A) [38]. The CAT levels were approximately 1000-fold lower than those produced by a CMV-promoter driven CAT plasmid named pCMVCAT16 (Fig 1A) that served as a positive control (Fig 1C). Mutational inactivation of splicing silencers downstream of late L1 splice site SA5639 as in pBELMCATenhanced CAT production (Fig 1C), as described previously [18]. We speculated that these reporter plasmids could be used to study the splicing enhancer at HPV-16 SA3358 andtherefore mutationally inactivated all 10 clusters of predicted ASF/SF2 sites in pBELMCAT, resulting in pTEx4MCAT (Fig 1A). These mutationsshould preventsplicing to SA3358 and redirected splicing to SA5639 [18], and should therefore cause a significant increase in CAT expression.Poor enhancer function at SA3358 yields high CAT production and vice versa. As can be seen from Figure 1C, pTEx4MCAT produced approximately 10-fold higher CAT levels than pBELMCAT, suggesting that splicing was redirected from SA3358 to SA5639. Mutational inactivation of the enhancer only, in the absence of inactivation of the silencer at the L1 3′-splice site SA5639 in pTEx4CAT,also resulted in a 10-fold increase in CAT productioncompared to pBELCAT (Fig C). All transfections were performed in triplicates and mean values and standard deviations are shown. Each plasmid was analysed in at least three independent transfection experiments. These results validated the pBELMCAT plasmid as a tool to study the splicing enhancer at HPV-16 SA3358.

To confirm that the introduction of mutations in the enhancer at SA3358, caused a skipping of the internal exon between SA3358 and SD3632, we also performed RT-PCR on RNA extracted from HeLa cells transfected with the various pBELCAT-derived plasmids. The results revealed that pBELMCAT produced both L1 and L1i mRNAs, and that the L1 mRNA that contained the internal exon between SA3358 and SD3632 dominated, as expected (Fig 1D) [38]. In contrast, pTEx4MCAT, in which all predicted ASF/SF2 binding sites downstream of SA3358 in pBELMCAThad been mutationallyinactivated, produced high levels of primarily L1i mRNAs as a result of skipping of the internal exon between SA3358 and SD3632 (Fig 1D) [18], [32].Plasmid pTex4CAT produced relatively low levels of L1 and L1i mRNAs, as expected (Fig 1D). A real time PCR assay that monitored L1 and L1i mRNA levels as described in Materials and Methods revealed that pTEx4MCAT produced L1i mRNA levels that were 5.8 fold higher than the L1i mRNA levels produced by pBELMCAT (Fig 1E). As expected, the mutations in the SA3358 enhancer in pTEx4MCAT resulted in lower levels of E4 mRNAs being produced from pTEx4MCAT than from pBELMCAT (Fig 1E).Similar results were obtained by quantitation of E4 and L1i mRNAs produced by pBELCAT and pTEx4CAT (see below). To further demonstrate that the SA3358 enhancer was independent of the mutations in L1, we cotransfectedpBELCAT with plasmid pASFDRS that expresses a negative ASF/SF2 mutant that has been shown to negatively interfere with the enhancer at SA3358 [18], [32]. Real time PCR on E4 mRNA produced by pBELCAT in the absence or presence of pASFDRS revealed that E4 mRNA levels dropped 4.9-fold (Fig 1G), an effect similar to the introduction of the mutations in the SA3358 enhancer (Fig 1F). We concluded that the CAT reporter assay could be used to characterise the enhancer downstream of SA3358.

### Location of a splicing enhancer in the HPV E2/E4 coding region is conserved among HPVs

**Figure 2:**
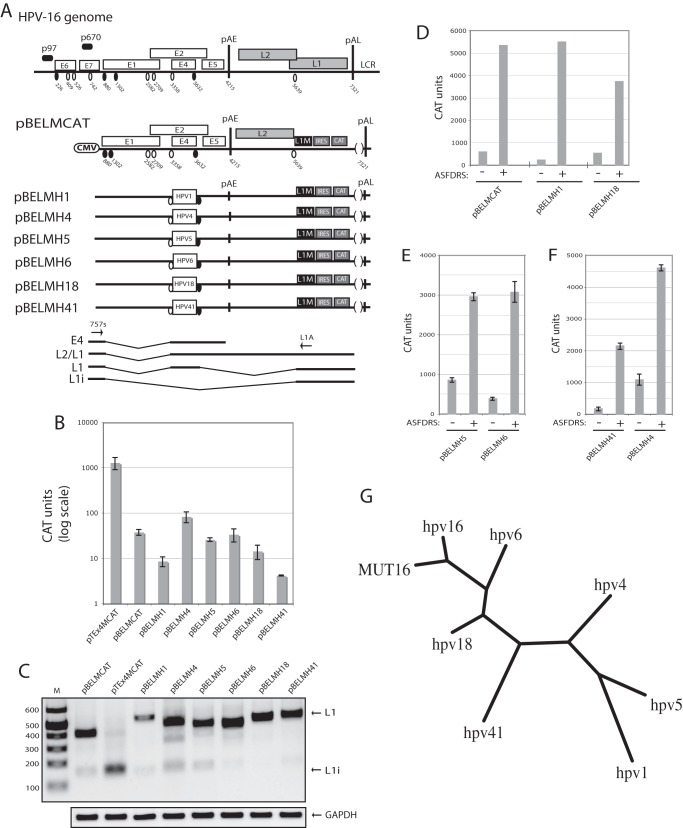
Location of a splicing enhancer in the HPV E2/E4 coding region is conserved among HPVs. (**A**). Schematic representation of the HPV-16 genome, the subgenomic HPV-16 expression plasmids and the control plasmid pCMVCAT16 [39]. The early and late viral promoters p97 and p670 are indicated. Numbers indicate nucleotide positions of 5′- (filled circles) and 3′-splice sites (open circles) or the early and late poly (A) sites pAE and pAL, respectively, and the borders of deletions. L1M represents a previously described mutant HPV-16 L1 sequence in which a number of nucleotide substitutions that inactivate splicing silencers have been inserted downstream of SA5639. Boxes indicate position of various HPV sequences inserted between nucleotide positions 3407 and 3627 in HPV-16 plasmid pBELMCAT [38]. IRES, the poliovirus internal ribosome entry site sequence; CAT, CAT reporter gene; CMV, human cytomegalovirus immediate early promoter; LCR, long control region. mRNAs produced by the plasmids are indicated. The positions of the RT-PCR primers (arrows) are indicated. (**B**) CAT protein levels produced in HeLa cells transfected with the indicated plasmids. CAT was monitored as described in Materials and Methods. Mean values and standard deviations are shown. Note the logarithmic scale. (**C**) RT-PCR with primers 757s and L1A on cDNA of cytoplasmic RNA extracted from HeLa cells transfected with the indicated plasmids. L1 and L1i mRNAs are indicated. M, molecular weight marker; GAPDH, cDNA amplified as internal control. (**D, E, F**) CAT protein levels produced in HeLa cells transfected with the indicated HPV plasmids in the presence or absence of ASFDRS expression plasmid [18]. CAT was monitored as described in Materials and Methods. Mean values and standard deviations are shown. Note that results in D and E are from transfections of cells in 24-well plates, whereas results in B and C are from 60 mm plates. (**G**) Phylogenetic tree of exonic sequences located between the splice sites that correspond to HPV-16 SA3358 and SD3632 of various HPV types. MUT16 represents the mutated HPV-16 sequence shown in Figure 1B.

The 3′-splice site that corresponds to HPV-16 SA3358in other HPV types,all conform poorly to a consensus 3′-splice, with the exception of HPV-4 (Table 1). This is mainly due to the absence of a long, uninterrupted polypyrimidine tract. One may speculate that the presence of a strong splicing enhancer downstream of the 3′-splice site that corresponds to SA3358 in HPV-16 is a conserved property of HPVs. To test this idea, we identified the “E4-exon” in the middle part of the genome in at least one HPV type of each genus of the *Papillomaviridae* family that contains a human papillomavirus. The exonic sequences from the various HPV types were inserted between SA3358 and SD3632 in the HPV-16 reporter plasmid pBELMCAT (Fig 2A) [38]. As described above, a strong enhancer downstream of SA3358 inhibits CAT, whereas a weak enhancer allows high CAT production due to skipping of SA3358 and alternative splicing to SA5639. The results revealed that all HPV types, except HPV-4, encoded enhancers of similar strength, or stronger than the HPV-16 enhancer since many of the plasmids produced less CAT than the HPV-16 plasmid pBELMCAT (Fig 2B). None of the plasmids produced more CAT than pTEx4MCAT in which the HPV-16 splicing enhancer had been mutationally inactivated. RT-PCR revealed that all plasmids produced late mRNAs of primarily the L1 type and not L1i, further supporting the conclusion that the various HPV sequences all directed splicing to the suboptimal 3′-splice site SA3358, although the HPV-4 sequence did so to a lower extent, as low levels of L1i mRNAs were produced from plasmid pBELMH4 (Fig 2C). The additional weak band running below the HPV-4 L1 mRNA is generated by a cryptic splice site in the HPV-4 sequence. It represents less than 1% of the L1 mRNAs produced from pBELMH4 and therefore does not interfere with the interpretation of the results.We concluded that the presence of a splicing enhancer in the “E4-exon” is a conserved property of HPVs.

**Table 1.Sequences:** of the HPV 3′splice site that corresponds to HPV-16 SA3358.

TYPE	GENUS (species)	3′- SPLICE SITE	PPT (nt)*
HPV-1	Mu	gtttacaaatgttatgtcttccact**AG**	6
HPV-4	Gamma	ccaagttatttcctcccctattgtt**AG**	11
HPV-5	Beta	ggaaactgtgtttgctcctgtcacc**AG**	5
HPV-6	Alpha (10)	tatatgttctcctgcatctgtatct**AG**	7
HPV-16	Alpha (9)	aatattatgtcctacatctgtgttt**AG**	4
HPV-18	Alpha (7)	aattgattgtaatgactctatgtgc**AG**	4
HPV-41	Nu	acctgaacctgtaactgttaccgac**AG**	3

* pyrimidines upstream of the invariable AG of the 3′-splice site are underlined.

We have previously shown that production of moderate levels of a mutant ASF/SF2 protein named ASFDRS, which binds RNA, but lacks the activation domain, functions as a trans-dominant mutant that inhibits the enhancer downstream of SA3358 in HPV-16 and causes alternative splicing to SA5639 [18]. However, increasing the ASFDRS levels further, inhibited also SA5639 and caused a total reduction of late HPV-16 mRNAs [18]. We therefore predicted that transfection of pBELMCAT with serially diluted ASFDRS plasmid should cause an induction of CAT production when low levels of ASFDRS plasmid are transfected.The results in Figure 2D show that ASFDRS increases CAT production from pBELMCAT. ASFDRS had a similar effect on plasmids encoding the HPV-1, HPV-4, HPV-5, HPV-6,HPV-18 and HPV-41 enhancers (Fig 2D-F), demonstrating that the presence of an ASF/SF2-dependent splicing enhancer in the “E4-exon” is a conserved property among low-risk and high-risk mucosal HPV types, as well as cutaneous HPV types. However, the enhancer is very loosely conserved, or consists of a very short sequence, as the HPV-16 exon sequence located between SA3358 and SD3632 is more closely related to the full HPV-16 mutant sequence in pTEx4M, than to the corresponding sequence in other HPV types (Fig 2G). These results prompted us to perform a mutational analysis of the enhancer located between splice sites SA3358 and SD3632 in the HPV-16 genome.

### One of the predicted ASF/SF2 binding sites downstream of HPV-16 SA3358 accounts for the majority of the enhancer activity

**Figure 3:**
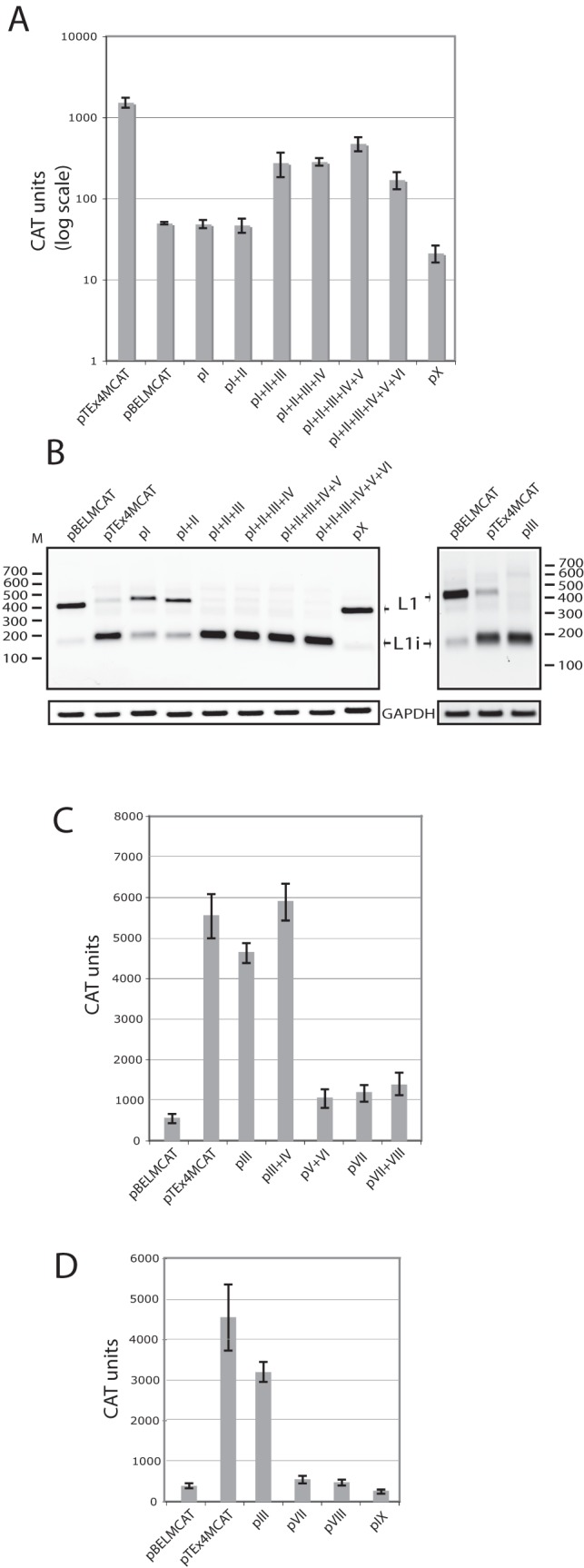
One of the predicted ASF/SF2 binding sites downstream of HPV-16 SA3358 accounts for the majority of the enhancer activity. (**A**). CAT protein levels produced in HeLa cells transfected with the indicated mutant pBELMCAT-derived plasmids [38]. CAT was monitored as described in Materials and Methods. Mean values and standard deviations are shown. Note the logarithmic scale. (**B**) RT-PCR with primers 757s and L1A on cDNA of cytoplasmic RNA extracted from HeLa cells transfected with the indicated plasmids. L1 and L1i mRNAs are indicated. M, molecular weight marker; GAPDH, cDNA amplified as internal control. (**C, D**) CAT protein levels produced in HeLa cells transfected with the indicated mutant pBELMCAT-derived plasmids [38]. CAT was monitored as described in Materials and Methods. Mean values and standard deviations are shown.

In order to determine the contribution of each predicted ASF/SF2 siteto the HPV-16 enhancer activity, we introduced mutations from site I (Fig 1B) in a consecutive manner, thereby creating plasmids pI, pI+IIetc in which predicted ASF/SF2 site I or site I and II were mutated, respectively. The numbering of the predicted ASF/SF2 sites is shown in Figure 1B and the names of mutant plasmids and mutated ASF/SF2 sites are shown in Table 2. Transfections of these plasmids into HeLa cells revealed that CAT production was largely unaffected by mutations in sites I and IIas plasmids pI and pI+II produced CAT levels similar to those produced by pBELMCAT (Fig 3A). When alsosite III was mutated, as in plasmid pI+II+III, CAT levels were substantially higher than those produced bypBELMCAT (Fig 3A). Mutations in sites IV and V further enhanced CAT production, but to a lower extent (Fig 3A). Introduction of mutations also in site VI, resulted in a reduction of CAT production (Fig 3A). Collectively, the results suggested that sites III, IV and V, contributed to the activity of the HPV-16 splicing enhancer downstream of SA3358, and that site III appeared to be of particular importance.

**Table 2:** Predicted ASF/SF2 sites that are mutated in the various HPV-16 reporter plasmids.

Plasmid name	ASF/SF2 sites in the enhancer that are mutated.
pTEx4M	I, II, III, IV, V, VI, VII, VIII, IX, X
pI	I
pI+II	I, II
pI+II+III	I, II, III
pI+II+III+IV	I, II, III, IV
pI+II+III+IV+V	I, II, III, IV, V
pI+II+III+IV+V+VI	I, II, III, IV, V, VI
pIII	III
pIII+IV	III, IV
pV+VI	V, VI
pVII	VII
pVII+VIII	VII, VIII
pVIII	VIII
pIX	IX
pX	X

If this conclusion was correct, all pBELMCATplasmids which contained mutations in site III should produce elevated levels of late mRNAs of L1i type, whereas pBELMCAT and mutants in which site III was unaffected such as pI, pII and pX, should produce low levels of late mRNAs of the L1typethat contain the internal exon between SA3358 and SD3632. The RT-PCR of the late L1 mRNAs shown in Figure 3B, demonstrated that all pBELMCAT-derived plasmids in which site III had been mutated produced high levels of L1i mRNAs (pTEx4MCAT, pI+II+III, pI+II+III+IV and pI+II+III+IV+V) with the exception of plasmid pI+II+III+IV+V+VI in which also site six was mutated and slightly lower levels of CAT were observed (Fig 3A and B). These results supported the conclusion that inactivation of site III caused an induction of L1i mRNA production as a result of skipping of the exon between SA3358 and SD3632.

To confirm these results, we mutated site III only in pBELMCAT, resulting in pIII (Table 2). Transfection of this plasmid revealed that pIIIproduced high levels of CAT (Fig 3C). As a matter of fact, double mutant pIII+IV (Table 1) produced CAT levels in the range of those produced by pTEx4MCAT (Fig. 3C), demonstrating that the enhancer had been inactivated by these mutations. Mutational inactivation of sites V+VI, VII+VIII, or site VII, VIII or IX alone, had a relatively small effect on CAT production, if any (Fig 3C and D), and mutational inactivation of site III stood out as the single mutation with the biggest effect on CAT production. Analysis of pIII by RT-PCR confirmed that primarily L1i mRNAs was produced (Fig 3B). We concluded that ASF/SF2 site III accounted for the majority of the activity of the HPV-16 splicing enhancer downstream of SA3358.

### Identification of single nucleotide substitutions that interfere with the activity of the HPV-16 splicing enhancer at SA3358

**Figure 4:**
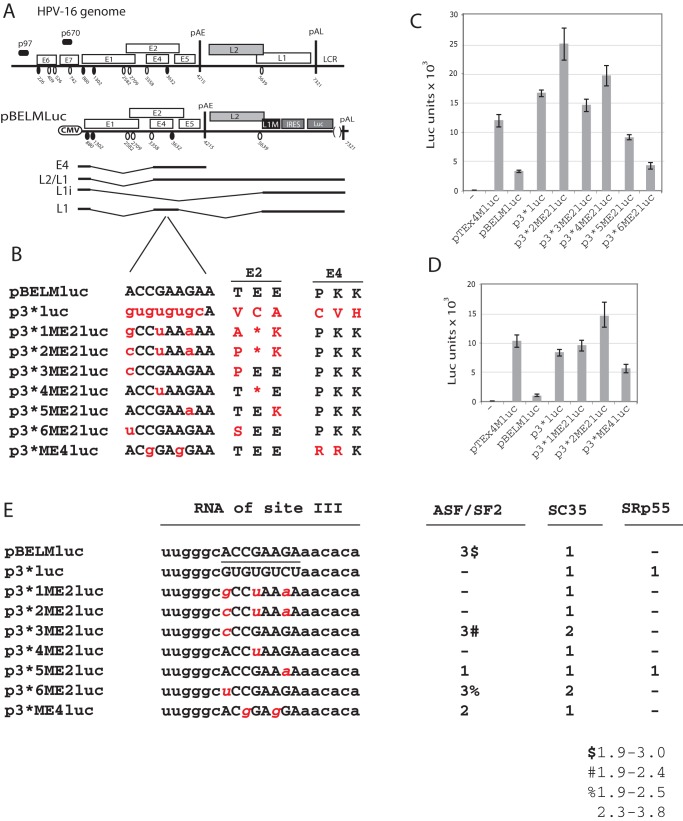
Identification of single nucleotide substitutions that interfere with the activity of the HPV-16 splicing enhancer at SA3358. (**A**). Schematic representation of the HPV-16 genome andsubgenomic HPV-16 expression plasmids. The early and late viral promoters p97 and p670 are indicated. Numbers indicate nucleotide positions of 5′- (filled circles) and 3′-splice sites (open circles) or the early and late poly (A) sites pAE and pAL, respectively, and the borders of deletions. L1M represents a previously described mutant HPV-16 L1 sequence in which a number of nucleotide substitutions that inactivate splicing silencers have been inserted downstream of SA5639. IRES, the poliovirus internal ribosome entry site sequence; luc, luciferase reporter gene; CMV, human cytomegalovirus immediate early promoter; LCR, long control region. mRNAs produced by the plasmids are indicated. (**B**) Sequences below plasmid maps represent wild type HPV-16 ASF/SF2 site III (capitals, black) and nucleotide substitutions (lower case, red) in the various plasmids indicated to the left.The effects of the nucleotide substitutions on the E2 and E4 protein sequences are indicated to the right. (**C, D**) Luciferase enzyme activity (Luc) produced in HeLa cells transfected with the indicated plasmids monitored as described in Materials and Methods. Mean values and standard deviations are shown. (**E**) Number and quality of binding sites of ASF/SF2, SC35 and SRp55 in the wild type and mutant sequences shown in (B) predicted by ESE finder.

The mutational inactivation of predicted ASF/SF2 binding site III described above consisted of eight nucleotide substitutions (Fig 4A and B). To investigate if the enhancer could be inactivated by fewer substitutions, we mutated this site byone to three nucleotide substitutions at a time. These sets of mutants were generated in pBELMluc in which the CAT reporter gene in pBELMCAT [38] had been replaced by the luciferase gene (Fig 4A). Analysis of pBELMluc and p3*luc (that contains the same enhancer mutation in predicted ASF/SF2 site III as CAT reporter plasmid pIII (Fig 4B), revealed that mutational inactivation of entire site III resulted in a 5-fold increase in luciferase levels (Fig 4C), similar to the results described above for the CAT plasmids pBELMCAT and pIII. This validated the Luciferase reporter plasmids. As the enhancer is located in a region of the HPV-16 genome in which both E2 and E4 open reading frames (orfs) overlap the enhancer, it is impossible to mutate the enhancer without also affecting the coding sequence of either E2 or E4. For example, the eight-nucleotide substitutions in predicted ASF/SF2 binding site III in p3*luc changed three amino acids in E2 and three amino acids in E4, including the addition of a termination codon (Fig 4A and B). However, we have shown that mutational inactivation of the E2 or E4 genes does not affect late gene expression from these reporter plasmids (Fig S1C), but they could potentially affect subsequent analysis of the enhancer in the context of the HPV-16 genome. The next set of enhancer mutations were therefore designed to affect either the E2 orf or the E4 orf. We first generated mutants that only affected the E2 orf named p3*1ME2luc to p3*6ME2luc, or the E4 orf, named p3*ME4luc (Fig 4B).Plasmids p3*1ME2luc and p3*2ME2luc produced high levels of luciferase, similar to those produced by p3*luc (Fig 4C and D), whereas p3*ME4luc displayed an intermediate phenotype (Fig 4D). We concluded that the mutations in p3*1ME2luc and p3*2ME2luc had the greatest effect on the splicing enhancer. Single nucleotide substitutions in site III of the enhanceras inp3*3ME2luc, p3*4ME2luc, p3*5ME2luc and p3*6ME2luc (Fig 4B), also induced luciferase production (Fig 4C and D), but to a lower extent than those in p3*1ME2luc and p3*2ME2luc. These resultsindicated that the enhancer was sensitive to small changes such as single nucleotide substitutions. A selection of the mutations was also introduced into a pBELM-derived plasmid with secreted luciferase (sLuc) as reporter gene replacing luc, with similar results (Fig S1). We also analysed the wild type and mutant ASF/SF2 site III sequences in ESE finder (Fig 4E) [43]. These results indicated that there was a correlation between number and quality of predicted ASF/SF2 binding sites in the various mutantsand luciferase production (Fig 4E). No correlation was seen with predicted binding of SR-proteins SC35 or SRp55. In conclusion, these results demonstrated that the splicing enhancer downstream of HPV-16 SA3358 was negatively affected by single nucleotide substitutions and that the same mutations were predicted to interfere with binding of ASF/SF2.

### ASF/SF2 binds to site III in the splicing enhancer at HPV-16 SA3358

**Figure 5:**
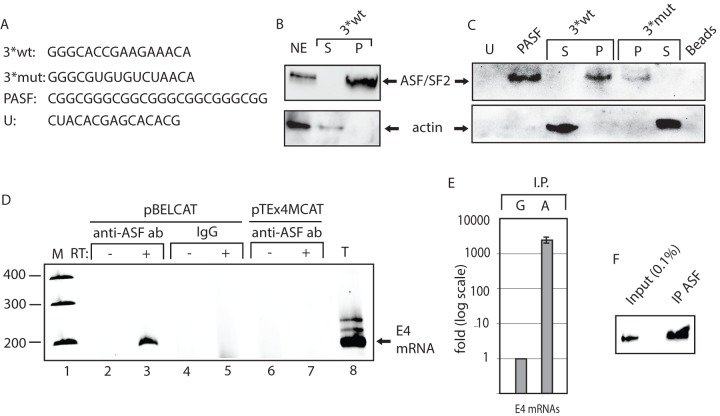
ASF/SF2 binds to site III in the splicing enhancer at HPV-16 SA3358. (**A**)**.** Sequences of the HPV-16 wild type (3*wt) and mutant (3*mut) enhancer site III sequence, four copies of an optimal ASF/SF2 binding site (PASF) serving as positive control and an unrelated RNA sequence termed “U”.The U sequence is adjacent to the ASF/SF2 site III in the HPV-16 genome. Biotinylated 2′-OMe-RNA forms of these sequences were used for RNA-protein pull-down experiments. (**B**) RNA-mediated pull down of ASF/SF2 from nuclear extracts protein usingbiotinylated, 2′-OMe-RNA sequences of wild type 3*wt RNA followed by Western blot analysis of RNA binding proteins in the pellet (P) or unbound proteins in the supernatant (S). (**C**) Western blot analysis of ASF/SF2 or actin after pull down of proteins using the various RNAs indicated in the figure. Pellet (P) represent the fraction of RNA bound proteins while unbound proteins are located in the supernatant (S). (**D**) RT-PCR on E4 mRNAs with primers 757s and E4A on cDNA synthesised from in vivo UV cross-linked, RNA-protein complexes immunoprecipitated with monospecific antibodies against ASF/SF2 (anti-ASF ab) or mouse IgG (IgG). Cells where either transfected with pBELCAT or the enhancer mutant pTEx4MCAT. RT-PCR was performed in the absence (−) or presence (+) of RT-enzyme for each sample. M, molecular weight markers; T, total cytoplasmic RNA. (**E**) Real time PCR of HPV-16 E4 mRNAs immunoprecipitated with mouse IgG (G) or anti-AFS/SF2 antibody (A) using primers 757s and E4A as described in Materials and Methods. (**F**) Western blot on ASF/SF2 immunoprecipitated by the murine anti-ASF/SF2 antibody revealed that approximately 2.3% of the ASF/SF2 proteins were immunoprecipitated.

To demonstrate interactions between site III and ASF/SF2, 2′-OMe-RNA of 3*wt or 3*mut (Fig 5A) with a biotin at the 5′-end were used in a pull down assays of ASF/SF2 from HeLa cell nuclear extract. Western blot of the proteins pulled down by the RNAs revealed that ASF/SF2 was found primarily in the pellet bound to 3*wt RNA, while actin was unbound and located primarily in the supernatant (Fig 5B). Further experiments showed that ASF/SF2 bound more efficiently to the wild type site III RNA sequence (3*wt)than to the mutantsite III RNA sequence (3*mut) (Fig 5C). ASF/SF2 bound strongly to a positive control RNA consisting of four repeated ASF/SF2 binding sites named PASF (Fig 5A and C),while little binding was observed using an unrelatedRNA sequences named “U” (Fig 5A and C). We concluded that ASF/SF2 binds more efficiently to the wild type than to the mutant enhancer sequence.

We also investigated if ASF/SF2 interacted with to the HPV-16 mRNA spliced from SD880 to SA3358 in living cells, and therefore transfected 293T cells with pBELluc and subjected the cells to UV cross linking, followed by cell lysis and immunoprecipitation with antibodies that specifically recognisedASF/SF2. RNA extracted from the precipitates was subjected to RT-PCR with primers 757S and E4A (see Fig 1A for primer location) that detectHPV-16 mRNAs that are spliced from SD880 to SA3358. As can be seen, spliced HPV-16 E4 mRNA was pulled down by the ASF/SF2 antibodybut not by murine IgG (Fig 5D), demonstrating that ASF/SF2 binds HPV-16 mRNAs in living cells. Real time PCR revealed that >3000-fold more HPV-16 E4 mRNAs were detected with the ASF/SF2 antibody than with murine IgG (Fig 5E). In contrast, GAPDH mRNAs were not immunoprecipitated by the anti-ASF/SF2 antibody (data not shown). We concluded that ASF/SF2 binds to HPV-16 mRNAs spliced from SD880 to SA3358 in living cells and that ASF/SF2 binds primarily to site III in the splicing enhancer.

### Predicted ASF/SF2 binding site III of the splicing enhancer at HPV-16 SA3358 is active in human primary keratinocytes

**Figure 6:**
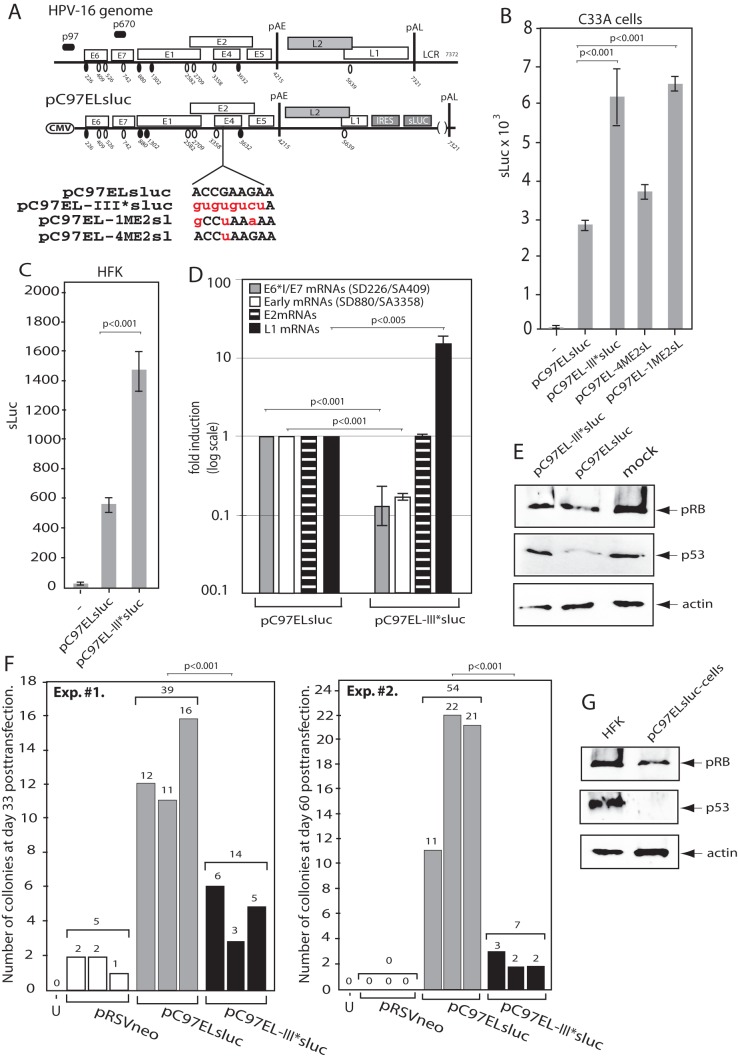
Mutational inactivation of ASF/SF2 binding site III in the HPV-16 splicing enhancer at SA3358 reduced the ability of HPV-16 to increase the life span of primary human keratinocytes. (**A**). Schematic representation of the HPV-16 genome and subgenomic HPV-16 expression plasmids. The early and late viral promoters p97 and p670 are indicated. Numbers indicate nucleotide positions of 5′- (filled circles) and 3′-splice sites (open circles) or the early and late poly (A) sites pAE and pAL, respectively, and the borders of deletions. IRES, the poliovirus internal ribosome entry site sequence; sLuc, secreted luciferase reporter gene; CMV, human cytomegalovirus immediate early promoter; LCR, long control region. Sequences below plasmid maps represent wild type HPV-16 ASF/SF2 site III (capitals, black) and nucleotide substitutions (lower case, red) in the various plasmids indicated to the left. (**B**) Luciferase enzyme activity (Luc) produced in C33A cells transfected with the indicated plasmids and monitored as described in Materials and Methods. Mean values and standard deviations are shown. (**C**) Luciferase enzyme activity (Luc) produced in primary human foreskin keratinocytes (HFK) cells transfected with the indicated plasmids and monitored as described in Materials and Methods. Mean values and standard deviations are shown. (**D**) Real time PCR on E6*I/E7 mRNAs spliced between HPV-16 splice sites SD226 and SA409 using primers E6S and 757 as, early mRNAs spliced between SD880 and SA3358 with 757s and E4A, E2 mRNAs using primers SD880 and E2A and HPV-16 L1 mRNAs using primers 757s and L1A was performed as described in Materials and Methods. (**E**) Western blot analysis of pRb, p53 and actin in HFKs transiently transfected with pC97ELsluc or pC97EL-III*sluc, or mock-transfected HFKs. (**F**) Two experiments (#1 and #2)showing the number of colonies observed 33 days or 60 days posttransfection of primary human foreskin keratinocytes (HFK) cells. Cells were transfected with pRSVneo alone or pRSVneo and pC97ELsluc (has wild type ASF/SF2 site III in the splicing enhancer downstream of SA3358) or pC97EL-III*sluc (has a mutant ASF/SF2 site III in the splicing enhancer downstream of SA3358). Transfected cells were incubated in G418 as described in Materials and Methods and colonies were counted as they appeared. Results from triplicates transfections are shown. (**G**) Western blot analysis of pRb, p53 and actin in an immortalised cell line created by transfection of primary human foreskin keratinocytes (HFK) with pC97ELsluc and in untransfected HFKs. U, untransfectedbut G418 treated cells.

In order to investigate if the splicing enhancer at HPV-16 SA3358 was active in the presence of all known HPV-16 splice sites, wemutatedASF/SF2 site III of the enhancer in plasmidpC97ELsluc (Fig 6A). The pC97ELsluc plasmid is driven by a CMV promoter in place of the early HPV-16 p97 promoter and therefore contains all known HPV-16 splice sites as well as all known HPV-16 genes, including E6 and E7. Mutational inactivation of ASF/SF2 site III in pC97ELsluc resulted in plasmid pC97EL-III*sluc (Fig 6A). Transfections of plasmidspC97ELsluc andpC97EL-III*slucinto C33A cellsrevealed that pC97EL-III*sluc produced more sluc than pC97ELsluc, as expected (Fig 6B). Similar results were obtained in HeLa cells (data not shown)and with pC97ELCAT and pC97EL-III*CAT that contain CAT reporter gene instead of sluc (Fig S1D). Introduction of a one-nucleotide substitution in ASF/SF2 binding site III as in pC97EL-4ME2sl (Fig 6A) did not substantially enhancesluc production (Fig 6B), but introduction of three substitutions as in pC97EL-1ME2sl (Fig 6A)induced sluc levels that were similar to those produced by the full site III mutantpC97EL-III*sluc (Fig 6B). Transfections of pC97ELsluc and pC97EL-III*sluc into human primary foreskin keratinocytes revealed that pC97EL-III*slucproduced higher sluc levels than pC97ELsluc and therefore confirmed the results obtained with C33A and HeLa cells (Fig 6C).Analysis of mRNAs produced in the transfected primary keratinocytes by real time PCR revealed that enhancer mutant plasmid pC97EL-III*slucproduced lower levels of E6*I/E7 mRNAs (mRNAs spliced between HPV-16 SD226 and SA409) than wild type plasmid pC97ELsluc, while the opposite was true for the L1 mRNAs (Fig 6D). This was reflected by a reduction of pRb and p53-levels in cells transiently transfected with pC97ELsluc but not in cells transfected with pC97EL-III*sluc (Fig 6E). While the reduction of pRB was relatively low, the effect on p53 was greater (Fig 6E). qPCR with primers spanning the SD880/SA3358- and the SD226/SA409-splice junctions yielded a similar reduction in mRNA levels, supporting the idea that all early mRNAs that are spliced to SA3358 are reduced to a similar extent when the SA3358 splicing enhancer is mutated (Fig 6D). In contrast, the levels of the E2 mRNAs which use another 3′-splice site, SA2709, were similar in pC97ELsluc-and pC97EL-III*sluc-transfected cells (Fig 6D).These results indicated that the enhancer was active in primary human keratinocytes. To provide further evidence for the activity of the enhancer in primary human keratinocytes, these cells were transfected with pTEx4Msluc and pBELMsluc, RNA was extracted and levels of E4 mRNAs and L1/L1i mRNAs were determined by real time PCR. As expected, wild type enhancer plasmid pBELMsluc produced higher E4 mRNA levels than pTEx4MCAT, while pTEx4MCAT produced higher levels of L1i mRNAs than pBELMsluc (data not shown). These results established thatthe SA3358 enhancer, and in particular predicted ASF/SF2 binding site III, wasactive in human primary keratinocytes.

### Mutational inactivation of ASF/SF2 binding site III in the HPV-16 splicing enhancer at SA3358 reduced the ability of HPV-16 to increase the life span of primary human keratinocytes

The most common HPV-16 E6 and E7 mRNAs are spliced fromSD880 to SA3358 [21], [22], [23], [31] and the results presented above revealed that enhancer mutant plasmid pC97EL-III*sluc produced less E6*I/E7 mRNAs than wild type plasmid pC97ELsluc. We thereforewished to investigate if mutations that compromised the SA3358 enhancer in the E6- and E7-encoding pC97ELsluc plasmidand reducedits ability to express E6 and E7, alsoaffected its ability to increase the life span of human primary keratinocytes. Since the vast majority of the E7, E6, E6*I, E6*II and E4 mRNAs are all spliced to SA3358, the levels of these mRNAs are reduced. As splicing between SD226 and SA409 is relatively efficient, it is reasonable to assume that relatively low levels of full-length E6 is produced in the transfected cells.The wild type pC97ELsluc and enhancer-mutant pC97EL-III*slucplasmids (Fig 6A) were transfected in triplicates with pRSVneo into primary keratinocytes, as described in Materials and Methods. The primary keratinocytes are grown in monolayer cultures and expanded under growth conditions that maintain poorly differentiated cells with properties similar to the cells in the basal layers of the epithelium. Anuntransfected control and a control transfected with pRSVneo plasmid only were always included. Appearing colonies were counted several weeks posttransfection when untransfected cells had died.The results obtained in two representative experiments are shown in Figure 6E. Experiment #1 was terminated at day 33 posttransfection and colonies were counted. As can be seen, a total number of 39 colonies were obtained from the three plates transfected with pC97ELsluc, 14 colonies with pC97EL-III*sluc and 5 with pRSVneo. Inexperiment #2, the transfected cells were maintained for 60 days, resulting in a total of 54colonies with pC97ELsluc,seven colonies with pC97EL-III*slucand none with pRSVneo (Fig 6F). Three of the clones obtained from thepC97ELsluc transfection are being passaged and have grown for 90 days posttransfectionwith no signs of crisis. Both pRB and p53 levels are reduced in this cell line compared to primary human keratinoocytes (HFKs) (Fig 6G). We concluded that mutations in the predicted ASF/SF2 binding site III in the SA3358 enhancernegatively affected expression of E6 and E7 mRNAs thereby reducing the ability of the subgenomic HPV-16 plasmids to increase the life span of primary human keratinocytes. These experiments demonstrate that the strength of the splicing enhancer at HPV-16 SA3358 affects the ability of HPV-16 to stimulate cell growth, thereby contributing to the pathogenic properties of HPV-16.

### Nucleotide substitutions in ASF/SF2 binding site III in the splicing enhancer at SA3358 inducedlate gene expression from the full-length,episomalform of the HPV-16 genome

**Figure 7:**
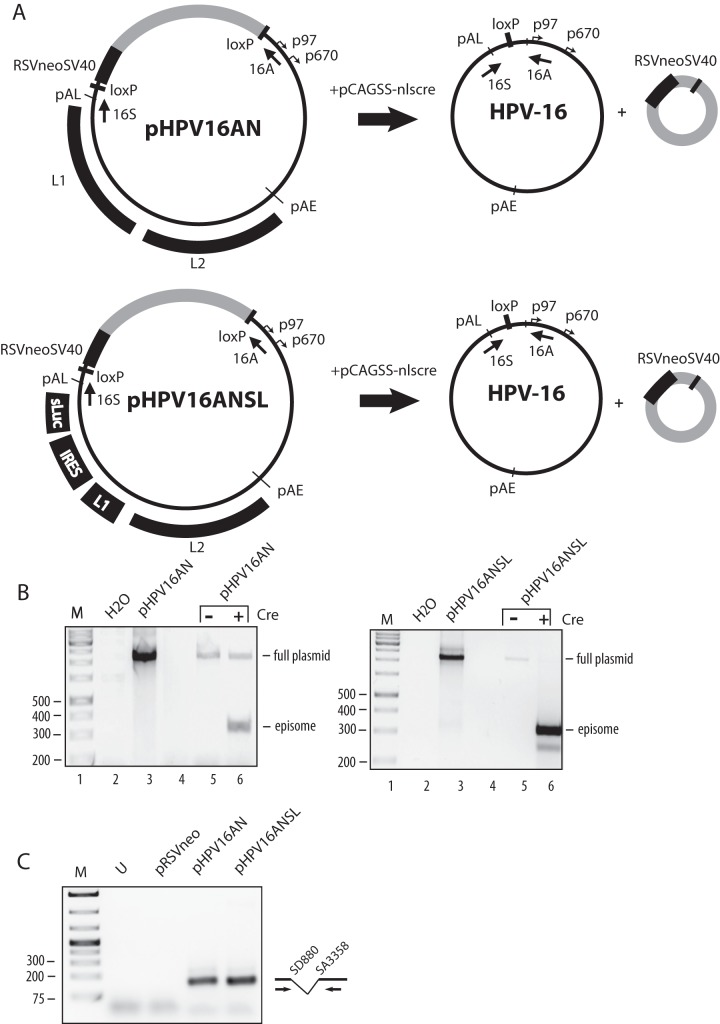
Generation of HPV-16 genomes with the secreted luciferase gene in place of L1. (**A**)**.** Structures of genomic HPV-16 plasmids pHPV16AN and pHPV16ANSL. LoxP sites and HPV-16 early (p97) and late (p670) promoters are indicated. Arrows denote positions of PCR primers 16S and 16A. The casette encoding the Rous sarcoma virus long terminal repeat promoter driving the neomycin resistance gene, followed by the simian virus 40 polyA signal is indicated. The effect of the crerecombinase on these plasmids is illustrated. pAE and pAL, HPV-16 early and late polyA signals, respectively; L1 and L2, late HPV-16 genes L1 and L2; sLuc, secreted luciferase; IRES, poliovirus 2A internal ribosome entry site. (**B**) PCR with primers 16S and 16A on total DNA extracted from human primary keratinocytes transfected with pHPV16AN or pHPV16ANSL in the absence or presence of the cre-expressing plasmid pCAGGS-nlscre (lanes 5 and 6) [37]. Primers 16S and 16A are located on each side of the LoxP sites in pHPV16AN and pHPV16ANSL and the PCR-reaction yields a 366-nucleotide PCR fragment that is diagnostic for recombination at the LoxP sites. A larger band is amplified from plasmid DNA that has not recombined (lane 3). A negative control is shown in lane 2. M, molecular size marker. (**C**) Nested RT-PCR on total RNA extracted from cells transfected with pHPV16AN, pHPV16ANsl or pRSVneo in the presence of pCAGGS-nlscre [37]. Primers 757s and E4A detect early HPV-16 mRNAs spliced from SD880 to SA3358 (E4), the most common early HPV mRNA. U, nested RT-PCR on RNA from untransfected cells; M, molecular size marker.

Next we wished to investigate if ASF/SF2 binding site III in the enhancer at SA3358 was active also in the context of the full HPV-16 genome. We therefore generated genomic plasmid pHPV16AN, in which a complete HPV-16 genome is flanked by loxP-sites and is released to its episomal form upon transfection of pHPV16AN with plasmid pCAGGS-nlscre [37], as previously described for HPV-18 (Fig 7A) [41]. In order to easily monitor and quantitate differences in HPV-16 late gene expression, we also generated pHPV16ANSL (Fig 7A), in which the L1 sequences from the BamHI site located 518 nucleotides downstream of the L1 ATG to the L1 stop codon were replaced by the poliovirus IRES followed by the sLuc reporter gene (Fig 7A). Effects of various mutations on HPV-16 late gene expression could then be determined by the quantitation of sLuc in the medium of the transfected cells. Human primary keratinocytes were transfected with pHPV16AN and pHPV16ANSL, in the absence or presence of pCAGGS-nlscre [37], and selected in G418 for 4 days before harvest of cells, as described in Materials and Methods. PCR-analysis of DNA extracted from the transfected cells, using primers flanking the recombination sites, revealed that recombination occurred and that episomal HPV-16 DNA formed in a pCAGGS-nlscre-dependent manner, as expected (Fig 7B). PCR primers were located on each side of the two LoxP sites, and PCR gave rise to a small band if recombination had occurred, as described previously for HPV-18 [41]. Extraction of RNA from the transfected cells followed by nested RT-PCR with primers that detect early mRNAs spliced from SD880 to SA3358, revealed that both plasmids produced similar levels of early mRNAs spliced to SA3358 (Fig 7C). We concluded that pHPV16ANSL could be used to monitor the effects of mutations in the splicing enhancer downstream of SA3358.

**Figure 8:**
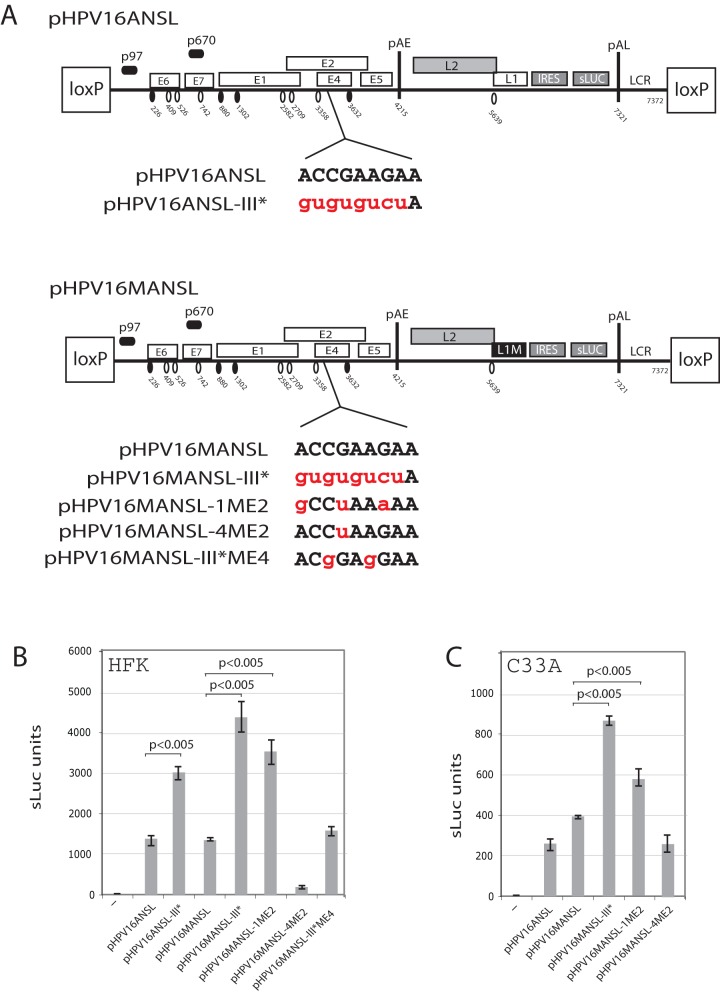
Nucleotide substitutions in ASF/SF2 binding site III in the splicing enhancer at SA3358 induced late gene expression from the full-length,episomalform of the HPV-16 genome. (**A**). Schematic representation of the HPV-16 genomic plasmids pHPV16ANSL and pHPV16MANSL. The early and late viral promoters p97 and p670 are indicated. Numbers indicate nucleotide positions of 5′- (filled arrow heads) and 3′-splice sites (open arrow heads) or the early and late poly (A) sites pAE and pAL, respectively. L1M represents a previously described mutant HPV-16 L1 sequence in which a number of nucleotide substitutions inactivate splicing silencer elements downstream of late 3′-splice site SA5639. IRES, the poliovirus internal ribosome entry site sequence; sLuc, secreted luciferase gene; LCR, long control region. Sequences below plasmid maps represent wild type HPV-16 ASF/SF2 site III (capitals, black) and nucleotide substitutions (lower case, red) in the various plasmids indicated to the left. (**B, C**). Cell culture medium ofhuman primary keratinocytes collected at day 5 posttransfection with the various indicated plasmids was subjected to secreted-luciferase assay as described in Materials and Methods. Transfections were performed in the presence of pCAGGS-nlscre. Mean values and standard deviations of sLuc activity in the cell culture med of triplicate transfections are shown.

We introduced mutations in the predicted ASF/SF2 binding site III in the full-length HPV-16 genomic plasmid pHPV16ANSL (Fig 8A), resulting in pHPV16ANSL-III* (Fig 8A). Inactivation of the splicing enhancer at SA3358 should enhance sluc expression as a result of redirection of splicing from SA3358 to SA5639 upstream of the L1 AUG. These plasmids were cotransfected with pCAGGS-nlscreinto human primary human foreskin keratinocytes (HFKs) followed by a 4 day selection in G418 before monitoring sLuc production.Indeed, introduction of eight nucleotide substitution in ASF/SF2 site number III in pHPV16ANSL-III*resulted in induction of late gene expression as measured by sluc (Fig 8B). Similar results were obtained when the mutations in the enhancer were inserted in combination with L1M as in plasmid pHPV16MANSL-III*, in which the splicing silencers at SA5639 were inactivated in order to enhance splicing to SA5639 (compare pHPV16MANSL and pHPV16MANSL-III*) (Fig 8B). Introduction of only three nucleotide substitutions in site III, as in pHPV16MANSL-1ME2, also caused a significant induction of late gene expression, whereas one or two substitutions as in pHPV16MANSL-4ME2 and pHPV16MANSL-III*ME4 did not (Fig 8B).Similar results were obtained by transfection into C33A cells, as expected (Fig 8C). We concluded that the single ASF/SF2 site affected the efficiency of splicing to HPV-16 3′-splice site SA3358 on mRNAs produced from the episomal form of the full-length HPV-16 genome.These results supported the conclusion that binding of ASF/SF2 the splicing enhancer downstream of SA3358 plays an important role in the HPV-16 gene expression program.

## Discussion

HPV-16 3′-splice site SA3358 is the most commonly used 3′-splice site on the HPV-16 genome [21], [22], [23], [31], yet it conforms very poorly to a consensus 3′-splice site that typically contains a long uninterrupted polypyrimidine tract upstream of the invariable AG dinucleotide [45]. Therefore, recognition of HPV-16 SA3358 requires a splicing enhancer [18], [2]. The advantage with this arrangement may be that SA3358 can be regulated by changes in levels of various splicing factors during the viral life cycle, in particular ASF/SF2, SRp30c and SRp20 [17], [18], [19]. This appears to be a conserved property among HPVs. First, exonic sequences located downstream of the 3′-splice site that corresponds to HPV-16 SA3358, obtained from 7 different HPV types belonging to 5 different genera, could replace the HPV-16 exonic splicing enhancer and direct splicing to HPV-16 SA3358. Second, the 3′-splice sites corresponding to HPV-16 SA3358 in the 7 HPV types from 5 different genera, all (with the possible exception of HPV-4) appeared to encode a weak 3′-splice site, with short, interrupted upstream polypyrimidine tracts (Table 2). Experiments performed on an infectious molecular clone of HPV-31 lend further support to the conclusion that a splicing enhancer in the “E4-region” is a conserved property of HPVs [46]. Mutational inactivation of HPV-31 SA3295, which corresponds to HPV-16 SA3358, activated a cryptic 3′-splice site located only three nucleotides further down [46], strongly suggesting the presence of a strong splicing enhancer in that region. One may speculate that it is an evolutionary advantage to regulate the utilisation efficiency of this 3′-splice site by the help of relative concentrations of cellular splicing factors, rather than encoding a 3′-splice site, which is constitutively active during the entire HPV life cycle.

At the early stage in the viral life cycle when primarily theHPV-16 early promoter p97 drives transcription, efficient splicing to SA3358 favoursexpression of the E6 and E7genes. The most common HPV E6 and E7 mRNAs are spliced between HPV-16 SD880 and SA3358 [20], [21], [22], [23], [31]. High levels of ASF/SF2 should therefore favour E6 and E7 expression, which is in line with the high expression levels of ASF/SF2 in the lower and middle layers of the cervical epithelium [35], [36]. In addition, HPV-16 infections that persist for decades and cause high-grade cervical lesions with risk of progressing to cancer and are stuck in the early stage of the viral life cycle with high E6 and E7 expression, also show elevated levels of SR-proteins, in particular ASF/SF2 [35], [36]. The ASF/SF2 protein was recently classified as a proto-oncogene [34], and ASF/SF2 may cooperate with HPV E6 and E7 to transform human keratinocytes. If high ASF/SF2-levels drive HPV-16 E6 and E7 expression, or if HPV-16 infection induces high ASF/SF2-production remains to be determined. High levels of ASF/SF2 would inhibit expression of E1 and E2 as they are expressed from partially- or alternatively spliced mRNAs that compete with the SA3358 splice site. In that respect it is interesting to note that SA3358 is negatively affected by overexpression of SRp20, SRp30c and ASFDRS [17], [18], [19]. Changes in the relative levels of ASF/SF2, SRp30c and SRp20 during epithelial cell differentiation, may therefore contribute to the temporal expression of the HPV-16 genes. The interplay between SRp20, SRp30c and ASF/SF2 in HPV-16 gene regulations is not fully understood.

Since the ESE finder [43] had predicted 10 clusters of fifteen ASF/SF2 binding sites within the HPV-16 exon between SA3358 and SD3632 [18], we were surprised to find that only one site contributed substantially to theenhancer activity.Consequently, the HPV-16 splicing enhancer at SA3358 will be sensitive to minor changes at this site. Our results showed that a single nucleotide substitution could reduce the activity of the enhancer. These results therefore suggested that nucleotide polymorphism at this site could affect the response of HPV-16 to ASF/SF2. It would therefore be of interest to investigate if this site in the HPV-16 genome varies between HPV-16 isolates, and if such variation correlates with disease progression. Furthermore, expression levels of ASF/SF2, SRp30c and SRp20 maydiffer between individuals, thereby affecting HPV-16 gene expression. Mutations in the ASF/SF2 site of the SA3358 splicing enhancer reduced E6 and E7 expression fromsubgenomic HPV-16 expression plasmids, thereby reducing their ability to stimulate cell proliferation and extending the life span of primary human keratinocytes. This is in line with previously reported results which showed that splicing to SA3358 was required for efficient expression of E6 and E7 [47]. Our results therefore link the strength of the splicing enhancer to the ability of HPV-16 to stimulate cell growth and induce immortalisation, i.e. to the pathogenic properties of HPV-16.

When the HPV-16 early promoter p97 is shut down and the late promoter p670 is induced, SA3358 continues to dominate among the HPV-16 splice sites. Efficient use of SA3358 results in high expression of E4, at the expense of E1 and E2. Since it has been shown that the expression levels of E2 and E4 change during differentiation [5], [6], [7], [48], one may speculate that the activity of the splicing enhancer at SA3358 also changes. After a state of high E1 and E2 production with efficient replication of the HPV DNA genome, high levels of E4 are normally produced followed by L1 and L2 production in the upper layers of the epithelium [5], [6], [7]. Since E4, L1 and L2 should all be favoured by efficient use of SA3358, it is reasonable to speculate that the HPV-16 infection causes an induction of ASF/SF2 as the cells differentiate. This induction of ASF/SF2 may be partly caused by E2 that has been shown to induce transcription of the ASF/SF2 gene [49].This would be in line with therecently described late function of E2, in which HPV-16 E2 induces HPV-16 late gene expression by inhibiting the viral early polyadenylation signal pAE [13]. Alternatively, other splicing factors that act on the SA3358 enhancer,for example SRp30c and SRp20 may regulate splicing to SA3358 in the upper layers of the epithelium. It would therefore be of interest to investigate if other factors bind the enhancer in terminally differentiated keratinocytes as opposed to mitotic cells, or cancer cells.

## Supporting Information

Figure S1 (A) Schematic representation of the HPV-16 genome and subgenomic HPV-16 expression plasmids. The early and late viral promoters p97 and p670 are indicated. Numbers indicate nucleotide positions of 5′- (filled circles) and 3′-splice sites (open circles) or the early and late poly (A) sites pAE and pAL, respectively, and the borders of deletions. L1M represents a previously described mutant HPV-16 L1 sequence in which a number of nucleotide substitutions that inactivate splicing silencers have been inserted downstream of SA5639. IRES, the poliovirus internal ribosome entry site sequence; sluc, secreted luciferase reporter gene; CMV, human cytomegalovirus immediate early promoter; LCR, long control region. mRNAs produced by the plasmids are indicated. (B) Secreted luciferase enzyme activity (sLuc units) produced in HeLa cells transfected with the indicated plasmids monitored as described in Materials and Methods. Mean values and standard deviations are shown. (C). Real time RT-PCR on total RNA extracted from cells transfected with pBELMCAT with wild type E2 and E4 genes pBELMCAT (E2+, E4+) and pBELMCAT with inactivated E2 and E4 genes (E2−, E4−). L1 and E4 mRNAs were monitored by primers 757s and L1A and 757s and E4A, respectively, as described in Materials and Methods. Similar levels of L1 mRNAs and E4 mRNAs were produced from the two plasmids. (D). Schematic representation of the subgenomic HPV-16 expression plasmids. Numbers indicate nucleotide positions of 5′- (filled circles) and 3′-splice sites (open circles) or the early and late poly (A) sites pAE and pAL, respectively. L1M represents a previously described mutant HPV-16 L1 sequence in which a number of nucleotide substitutions that inactivate splicing silencers have been inserted downstream of SA5639. IRES, the poliovirus internal ribosome entry site sequence; CAT, CAT reporter gene; CMV, human cytomegalovirus immediate early promoter; LCR, long control region. Wild type site III is displayed as capitals and mutant site III is red and in lower case.
(PDF)

Table S1 **List of plasmids.**
(DOCX)

Table S2 **List of primers.**
(DOCX)

## References

[1] zur Hausen H (2002) Papillomaviruses and cancer: from basic studies to clinical application. Nat Rev Cancer 2: 342–350. 12044010 10.1038/nrc798

[2] Howley PM, Lowy DR (2006) Papillomaviridae. In: Knipe DM, Howley PM, editors. Virology. 5 ed: Lippincott/The Williams & Wilkins Co. Philadelphia, Pa. 2299–2354.

[3] Walboomers JM, Jacobs MV, Manos MM, Bosch FX, Kummer JA, et al (1999) Human papillomavirus is a necessary cause of invasive cervical cancer worldwide. J Pathol 189: 12–19. 10451482 10.1002/(SICI)1096-9896(199909)189:1<12::AID-PATH431>3.0.CO;2-F

[4] Bulkmans NW, Berkhof J, Bulk S, Bleeker MC, van Kemenade FJ, et al (2007) High-risk HPV type-specific clearance rates in cervical screening. Br J Cancer 96: 1419–1424. 17342094 10.1038/sj.bjc.6603653 PMC2360183

[5] Chow LT, Broker TR, Steinberg BM (2010) The natural history of human papillomavirus infections of the mucosal epithelia. APMIS 118: 422–449. 20553526 10.1111/j.1600-0463.2010.02625.x

[6] Doorbar J (2005) The papillomavirus life cycle. J Clin Virol 32 Suppl 1S7–15. 15753007 10.1016/j.jcv.2004.12.006

[7] Moody CA, Laimins LA (2010) Human papillomavirus oncoproteins: pathways to transformation. Nat Rev Cancer 10: 550–560. 20592731 10.1038/nrc2886

[8] Bernard HU (2002) Gene expression of genital human papillomaviruses and considerations on potential antiviral approaches. Antiviral Ther 7: 219–237. 12553476

[9] Thierry F (2009) Transcriptional regulation of the papillomavirus oncogenes by cellular and viral transcription factors in cervical carcinoma. Virology 384: 375–379. 19064276 10.1016/j.virol.2008.11.014

[10] Graham SV (2008) Papillomavirus 3′ UTR regulatory elements. Front Biosci 13: 5646–5663. 18508613 10.2741/3107

[11] Zheng ZM, Baker CC (2006) Papillomavirus genome structure, expression, and posttranscriptional regulation. Front Biosci 11: 2286–2302. 16720315 10.2741/1971 PMC1472295

[12] Schwartz S (2008) HPV-16 RNA processing. Frontiers in Bioscience 13: 5880–5891. 18508629 10.2741/3123

[13] Johansson C, Somberg M, Li X, Backström Winquist E, Fay J, et al (2012) HPV-16 E2 contributes to induction of HPV-16 late gene expression by inhibiting early polyadenylation. EMBO J 13: 3212–3227. 10.1038/emboj.2012.147 PMC3400011 22617423

[14] Johansson C, Somberg M, Schwartz S (2010) Proteins involved in HPV-16 mRNA processing. Current Topics in Virology 8: 17–27.

[15] Oberg D, Fay J, Lambkin H, Schwartz S (2005) A downstream polyadenylation element in human papillomavirus type 16 encodes multiple GGG-motifs and interacts with hnRNP H. J Virol. 79: 9254–9269. 10.1128/JVI.79.14.9254-9269.2005 PMC1168734 15994820

[16] Terhune SS, Hubert WG, Thomas JT, Laimins LA (2001) Early polyadenylation signals of human papillomavirus type 31 negatively regulate capsid gene expression. J Virol 75: 8147–8157. 11483760 10.1128/JVI.75.17.8147-8157.2001 PMC115059

[17] Somberg M, Li X, Johansson C, Orru B, Chang R, et al (2011) SRp30c activates human papillomavirus type 16 L1 mRNA expression via a bimodal mechanism. J Gen Virol 92: 2411–2421. 21697349 10.1099/vir.0.033183-0

[18] Somberg M, Schwartz S (2010) Multiple ASF/SF2 sites in the HPV-16 E4-coding region promote splicing to the most commonly used 3′-splice site on the HPV-16 genome. J Virol 84: 8219–8230. 20519389 10.1128/JVI.00462-10 PMC2916536

[19] Jia R, Liu X, Tao M, Kruhlak M, Guo M, et al (2009) Control of the papillomavirus early-to-late switch by differentially expressed SRp20. J Virol 83: 167–180. 18945760 10.1128/JVI.01719-08 PMC2612334

[20] Baker C, Calef C (1997) Maps of papillomavirus mRNA transcripts. In: Billakanti SR, Calef CE, Farmer AD, Halpern AL, Myers GL, editors. Human papillomaviruses: A compilation and analysis of nucleic acid and amino acid sequences. Los Almos: Theoretical biology and biophysics, Los Alamos National Laboratory.

[21] Doorbar J, Parton A, Hartley K, Banks L, Crook T, et al (1990) Detection of novel splicing patterns in a HPV16-containing keratinocyte cell line. Virology 178: 254–262. 2167553 10.1016/0042-6822(90)90401-c

[22] Grassmann K, Rapp B, Maschek H, Petry KU, Iftner T (1996) Identification of a differentiation-inducible promoter in the E7 open reading frame of human papillomavirus type 16 (HPV-16) in raft cultures of a new cell line containing high copy numbers of episomal HPV-16 DNA. J Virol 70: 2339–2349. 8642661 10.1128/jvi.70.4.2339-2349.1996 PMC190076

[23] Smotkin D, Wettstein FO (1986) Transcription of human papillomavirus type 16 early genes in a cervical cancer and a cancer-derived cell line and identification of the E7 protein. Proc Natl Acad Sci USA 83: 4680–4684. 3014503 10.1073/pnas.83.13.4680 PMC323805

[24] Chow LT, Nasseri M, Wolinsky SM, Broker TR (1987) Human papillomavirus types 6 and 11 mRNAs from genital condylomata acuminata. J Virol 61: 2581–2588. 3037118 10.1128/jvi.61.8.2581-2588.1987 PMC255705

[25] Nasseri M, Hirochika R, Broker TR, Chow L (1987) A human papillomavirus type 11 transcript encoding and E1-E4 protein. Virology 159: 433–439. 2887066 10.1016/0042-6822(87)90482-x

[26] Rotenberg MO, Chow LT, Broker TR (1989) Characterisation of rare human papillomavirus type 11 mRNAs coding for regulatory and structural proteins, using the polymerase chain reaction. Virology 172: 489–497. 2552659 10.1016/0042-6822(89)90191-8

[27] Hummel M, Hudson JB, Laimins LA (1992) Differentiation-induced and constitutive transcription of human papillomavirus type 31b in cell lines containing viral episomes. JVirol 66: 6070–6080. 1326657 10.1128/jvi.66.10.6070-6080.1992 PMC241484

[28] Hummel M, Lim HB, Laimins LA (1995) Human papillomavirus type 31b late gene expression is regulated through protein kinase C-mediated changes in RNA processing. J Virol 69: 3381–3388. 7745684 10.1128/jvi.69.6.3381-3388.1995 PMC189050

[29] Milligan SG, Veerapraditsin T, Ahamet B, Mole S, Graham SV (2007) Analysis of novel human papillomavirus type 16 late mRNAs in differentiated W12 cervical epithelial cells. Virology 360: 172–181. 17098271 10.1016/j.virol.2006.10.012 PMC2151308

[30] Ozbun MA, Meyers C (1997) Characterization of late gene transcripts expressed during vegetative replication of human papillomavirus type 31b. J Virol 71: 5161–5172. 9188583 10.1128/jvi.71.7.5161-5172.1997 PMC191751

[31] Schmitt M, Dalstein V, Waterboer T, Clavel C, Gissman L, et al (2010) Diagnosing cervical cancer and high-grade precursors by HPV-16 transcription patterns Cancer Res. 70: 249–256. 10.1158/0008-5472.CAN-09-2514 20028865

[32] Rush M, Zhao X, Schwartz S (2005) A splicing enhancer in the E4 coding region of human papillomavirus type 16 is required for early mRNA splicing and polyadenylation as well as inhibition of premature late gene expression. J Virol 79: 12002–12015. 16140776 10.1128/JVI.79.18.12002-12015.2005 PMC1212645

[33] Long JC, Caceres JF (2009) The SR protein family of splicing factors: master regulators of gene expression. Biochem J 417: 15–27. 19061484 10.1042/BJ20081501

[34] Karni R, de Stanchina E, Lowe SW, Sinha R, Mu D, et al (2007) The gene encoding the splicing factor SF2/ASF is a proto-oncogene. Nat Struct Mol Biol 14: 185–193. 17310252 10.1038/nsmb1209 PMC4595851

[35] Mole S, McFarlane M, Chuen-Im T, Milligan SG, Millan D, et al (2009) RNA splicing factors regulated by HPV16 during cervical tumour progression. J Pathol 219: 383–391. 19718710 10.1002/path.2608 PMC2779514

[36] Fay J, Kelehan P, Lambkin H, Schwartz S (2009) Increased expression of cellular RNA-binding proteins in HPV-induced neoplasia and cervical cancer. J Med Virol 81: 897–907. 19319956 10.1002/jmv.21406

[37] Nagy A (2000) Cre recombinase: the universal reagent for genome tailoring. Genesis 26: 99–109. 10686599

[38] Orru B, Cunniffe C, Ryan F, Schwartz S (2012) Development and validation of a novel reporter assay for human papillomavirus type 16 late gene expression. J Virol Meth 183: 106–116. 10.1016/j.jviromet.2012.03.023 22484615

[39] Zhao X, Schwartz S (2008) Inhibition of HPV-16 L1 expression from L1 cDNAs correlates with the presence of hnRNP A1 binding sites in the L1 coding region. Virus Genes 36: 45–53. 18040766 10.1007/s11262-007-0174-0

[40] Zhao X, Öberg D, Rush M, Fay J, Lambkin H, et al (2005) A 57 nucleotide upstream early polyadenylation element in human papillomavirus type 16 interacts with hFip1, CstF-64, hnRNP C1/C2 and PTB. J Virol 79: 4270–4288. 15767428 10.1128/JVI.79.7.4270-4288.2005 PMC1061554

[41] Wang HK, Duffy AA, Broker TR, Chow LT (2009) Robust production and passaging of infectious HPV in squamous epithelium of primary human keratinocytes. Genes Dev 23: 181–194. 19131434 10.1101/gad.1735109 PMC2648537

[42] Dignam JD, Lebovitz RM, Roeder RG (1983) Accurate transcription initiation by RNA polymerase II in a soluble extract from isolated mammalian nuclei. Nucleic Acids Res 11: 1475–1489. 6828386 10.1093/nar/11.5.1475 PMC325809

[43] Cartegni L, Wang J, Zhu Z, Zhang MQ, Krainer AR (2003) ESEfinder: A web resource to identify exonic splicing enhancers. Nucleic Acids Res 31: 3568–3571. 12824367 10.1093/nar/gkg616 PMC169022

[44] Zhao X, Rush M, Schwartz S (2004) Identification of an hnRNP A1 dependent splicing silencer in the HPV-16 L1 coding region that prevents premature expression of the late L1 gene. J Virol 78: 10888–10905. 15452209 10.1128/JVI.78.20.10888-10905.2004 PMC521837

[45] Chen M, Manley JL (2009) Mechanisms of alternative splicing regulation: insights from molecular and genomics approaches. Nat Rev Mol Cell Biol 11: 741–754. 10.1038/nrm2777 PMC2958924 19773805

[46] Klumpp D, Stubenrauch F, Laimins LA (1997) Differential effects of the splice acceptor at nucleotide 3295 of human papillomavirus 31 on stable and transient viral replication. J Virol 71: 8186–8194. 9343169 10.1128/jvi.71.11.8186-8194.1997 PMC192275

[47] Belaguli NS, Pater MM, Pater A (1992) Nucleotide 880 splice donor site required for efficient transformation and RNA accumulation by human papillomavirus type 16 E7 gene. J Virol 66: 2724–2730. 1313897 10.1128/jvi.66.5.2724-2730.1992 PMC241027

[48] Xue Y, Bellanger S, Zhang W, Lim D, Low J, et al (2010) HPV16 E2 is an immediate early marker of viral infection, preceding E7 expression in precursor structures of cervical carcinoma. Cancer Res 70: 5316–5325. 20530671 10.1158/0008-5472.CAN-09-3789

[49] Mole S, Milligan SG, Graham SV (2009) Human papillomavirus type 16 E2 protein transcriptionally activates the promoter of a key cellular splicing factor, SF2/ASF. J Virol 83: 357–367. 18945764 10.1128/JVI.01414-08 PMC2612322

