# Supplementary material for: Eight Nucleotide Substitutions Inhibit Splicing to HPV-16 3′-Splice Site SA3358 and Reduce the Efficiency by which HPV-16 Increases the Life Span of Primary Human Keratinocytes
Source: PLoS One. 2013 Sep 9;8(9):e72776. doi: 10.1371/journal.pone.0072776 (PMC3767658; doi:10.1371/journal.pone.0072776)
Supplement: Figure S1 — (A) Schematic representation of the HPV-16 genome and subgenomic HPV-16 expression plasmids. The early and late viral promoters p97 and p670 are indicated. Numbers indicate nucleotide positions of 5′- (filled circles) and 3′-splice sites (open circles) or the early and late poly (A) sites pAE and pAL, respectively, and the borders of deletions. L1M represents a previously described mutant HPV-16 L1 sequence in which a number of nucleotide substitutions that inactivate splicing silencers have been inserted downstream of SA5639. IRES, the poliovirus internal ribosome entry site sequence; sluc, secreted luciferase reporter gene; CMV, human cytomegalovirus immediate early promoter; LCR, long control region. mRNAs produced by the plasmids are indicated. (B) Secreted luciferase enzyme activity (sLuc units) produced in HeLa cells transfected with the indicated plasmids monitored as described in Materials and Methods. Mean values and standard deviations are shown. (C). Real time RT-PCR on total RNA extracted from cells transfected with pBELMCAT with wild type E2 and E4 genes pBELMCAT (E2+, E4+) and pBELMCAT with inactivated E2 and E4 genes (E2−, E4−). L1 and E4 mRNAs were monitored by primers 757s and L1A and 757s and E4A, respectively, as described in Materials and Methods. Similar levels of L1 mRNAs and E4 mRNAs were produced from the two plasmids. (D). Schematic representation of the subgenomic HPV-16 expression plasmids. Numbers indicate nucleotide positions of 5′- (filled circles) and 3′-splice sites (open circles) or the early and late poly (A) sites pAE and pAL, respectively. L1M represents a previously described mutant HPV-16 L1 sequence in which a number of nucleotide substitutions that inactivate splicing silencers have been inserted downstream of SA5639. IRES, the poliovirus internal ribosome entry site sequence; CAT, CAT reporter gene; CMV, human cytomegalovirus immediate early promoter; LCR, long control region. Wild type site III is displayed as capitals and m [file pone.0072776.s001.pdf]

Figure S1.

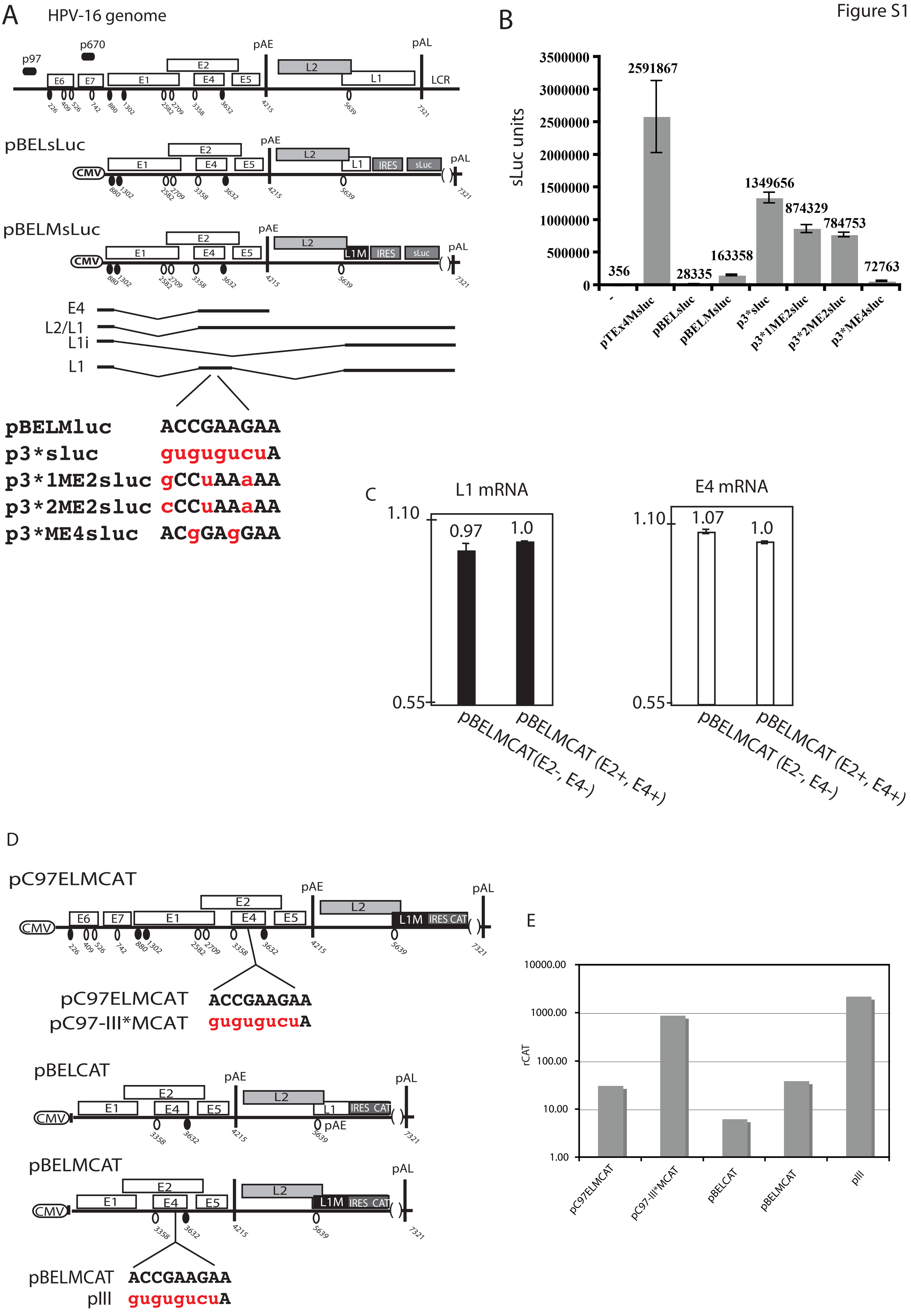
A
HPV-16 genome
p97
p670
pAE
pAL
E6
E7
E1
E2
E4
E5
L2
L1
LCR
pBELsLuc
CMV
IRES
sLuc
pBELMsLuc
L1M
E4
L2/L1
L1i
L1
pBELMluc ACCGAAGAA
p3*sluc gugugucuA
p3*1ME2sluc gCCuAAaAA
p3*2ME2sluc cCCuAAaAA
p3*ME4sluc ACgGAgGAA
B
sLuc units
356
2591867
28335
163358
1349656
874329
784753
72763
-
pTEx4Msluc
pBELsluc
pBELMsluc
p3*sluc
p3*1ME2sluc
p3*2ME2sluc
p3*ME4sluc
C
L1 mRNA
E4 mRNA
1.10
0.55
0.97
1.0
1.07
1.0
pBELMCAT(E2-, E4-)
pBELMCAT (E2+, E4+)
D
pC97ELMCAT
pC97ELMCAT ACCGAAGAA
pC97-III*MCAT gugugucuA
pBELCAT
IRES CAT
pBELMCAT
pBELMCAT ACCGAAGAA
pIII gugugucuA
E
rCAT
10000.00
1000.00
100.00
10.00
1.00
pC97ELMCAT
pC97-III*MCAT
pBELCAT
pBELMCAT
pIII
