# Supplementary material for: Eight Nucleotide Substitutions Inhibit Splicing to HPV-16 3′-Splice Site SA3358 and Reduce the Efficiency by which HPV-16 Increases the Life Span of Primary Human Keratinocytes
Source: PLoS One. 2013 Sep 9;8(9):e72776. doi: 10.1371/journal.pone.0072776 (PMC3767658; doi:10.1371/journal.pone.0072776)
Supplement: Table S1 — List of plasmids. (DOCX) [file pone.0072776.s002.docx]

**Table S1. List of plasmids**

| **Plasmid name** | **Source** | **Structure** | **Characteristics** | **References** |
| --- | --- | --- | --- | --- |
| pBELCAT | pBEL | Subgenomic HPV16 plasmid. CMV-promoter replacing HPV promoter at position 670 and IRES-CAT replacing L1 downstream of BamHI. Lacks late UTR NRE. | - | pBELCAT [38]  pBEL [42] |
| pTex4CAT | pBELCAT | - “ - | E4 enhancer mutant. |  |
| pBELMCAT | pBELM | Subgenomic HPV16 plasmid. CMV-promoter replacing HPV promoter at position 670 and IRES-CAT replacing L1 downstream of BamHI. Splicing silencer between SA5639 and BamHI in L1 is mutated. Lacks late UTR NRE. | E4 enhancer mutant. | pBELMCAT [38]  pBELM [42] |
| pTex4MCAT | pBELMCAT | - “ - | E4 enhancer mutant. |  |
| pBELMH1 | pBELMCAT | - “ - | E4 enhancer mutant. |  |
| pBELMH4 | pBELMCAT | - “ - | E4 enhancer mutant. |  |
| pBELMH5 | pBELMCAT | - “ - | E4 enhancer mutant. |  |
| pBELMH6 | pBELMCAT | - “ - | E4 enhancer mutant. |  |
| pBELMH18 | pBELMCAT | - “ - | E4 enhancer mutant. |  |
| pBELMH41 | pBELMCAT | - “ - | E4 enhancer mutant. |  |
| pI | pBELMCAT | - “ - | E4 enhancer mutant. |  |
| pI+II | pBELMCAT | - “ - | E4 enhancer mutant. |  |
| pI+II+III | pBELMCAT | - “ - | E4 enhancer mutant. |  |
| pI+II+III+IV | pBELMCAT | - “ - | E4 enhancer mutant. |  |
| pI+II+III+IV+V | pBELMCAT | - “ - | E4 enhancer mutant. |  |
| pI+II+III+IV+V+VI | pBELMCAT | - “ - | E4 enhancer mutant. |  |
| pIII+IV | pBELMCAT | - “ - | E4 enhancer mutant. |  |
| pV+VI | pBELMCAT | - “ - | E4 enhancer mutant. |  |
| pVII+VIII | pBELMCAT | - “ - | E4 enhancer mutant. |  |
| pIII | pBELMCAT | - “ - | E4 enhancer mutant. |  |
| pVII | pBELMCAT | - “ - | E4 enhancer mutant. |  |
| pVIII | pBELMCAT | - “ - | E4 enhancer mutant. |  |
| pIX | pBELMCAT | - “ - | E4 enhancer mutant. |  |
| pX | pBELMCAT | - “ - | E4 enhancer mutant. |  |
| pBELMluc | pBELMCAT | Subgenomic HPV16 plasmid. CMV-promoter replacing HPV promoter at position 670 and IRES-LUC replacing L1 downstream of BamHI. Splicing silencer between SA5639 and BamHI in L1 is mutated. Lacks late UTR NRE. | - |  |
| p3*luc | pBELMCAT | - “ - | E4 enhancer mutant. |  |
| p3*1ME2luc | pBELMCAT | - “ - | E4 enhancer mutant. |  |
| p3*2ME2luc | pBELMCAT | - “ - | E4 enhancer mutant. |  |
| p3*3ME2luc | pBELMCAT | - “ - | E4 enhancer mutant. |  |
| p3*4ME2luc | pBELMCAT | - “ - | E4 enhancer mutant. |  |
| p3*5ME2luc | pBELMCAT | - “ - | E4 enhancer mutant. |  |
| p3*6ME2luc | pBELMCAT | - “ - | E4 enhancer mutant. |  |
| p3*ME4luc | pBELMCAT | - “ - | E4 enhancer mutant. |  |
| pBELsluc | pBELCAT | Subgenomic HPV16 plasmid. CMV-promoter replacing HPV promoter at position 670 and IRES-sLuc replacing L1 downstream of BamHI. Lacks late UTR NRE. | - |  |
| pBELMsluc | pBELMCAT | Subgenomic HPV16 plasmid. CMV-promoter replacing HPV promoter at position 670 and IRES-sLuc replacing L1 downstream of BamHI. Splicing silencer between SA5639 and BamHI in L1 is mutated. Lacks late UTR NRE. | - |  |
| p3*sluc | pBELMCAT | - “ - | E4 enhancer mutant. |  |
| p3*1ME2sluc | pBELMCAT | - “ - | E4 enhancer mutant. |  |
| p3*2ME2sluc | pBELMCAT | - “ - | E4 enhancer mutant. |  |
| p3*ME4lsluc | pBELMCAT | - “ - | E4 enhancer mutant. |  |
| pC97ELsluc | pC97EL | Subgenomic HPV16 plasmid. CMV-promoter replacing HPV promoter at position p97 and IRES-sLuc replacing L1 downstream of BamHI. Lacks late UTR NRE. | - | pC97EL [43] |
| pC97EL-III*sluc | pC97EL | - “ - | E4 enhancer mutant. |  |
| pC97EL-1ME2sl | pC97EL | - “ - | E4 enhancer mutant. |  |
| pC97EL-4ME2sl | pC97EL | - “ - | E4 enhancer mutant. |  |
| pC97ELMCAT | pC79ELM | Subgenomic HPV16 plasmid. CMV-promoter replacing HPV promoter at position p97 and IRES-CAT replacing L1 downstream of BamHI. Splicing silencer between SA5639 and BamHI in L1 is mutated. Lacks late UTR NRE. | - |  |
| pC97ELM-III*CAT | pC79ELM | - “ - | E4 enhancer mutant. |  |
| pHPV16AN |  | Full HPV-16 genome flanked by SphI and loxP sites in a deleted pCRTOPO plasmid. | - |  |
| pHPV16ANSL | pHPV16AN | Full HPV-16 genome flanked by SphI and loxP sites in a deleted pCRTOPO plasmid. IRES-sLuc has replaced L1 downstream of BamHI. | - |  |
| pHPV16ANSL-III* | pHPV16AN | - “ - | E4 enhancer mutant. |  |
| pHPV16MANSL | pHPV16AN | Full HPV-16 genome flanked by SphI and loxP sites in a deleted pCRTOPO plasmid. IRES-sLuc has replaced L1 downstream of BamHI. Splicing silencer between SA5639 and BamHI in L1 is mutated. | - |  |
| pHPV16MANSL-III* | pHPV16AN | - “ - | E4 enhancer mutant. |  |
| pHPV16MANSL-1ME2 | pHPV16AN | - “ - | E4 enhancer mutant. |  |
| pHPV16MANSL-4ME2 | pHPV16AN | - “ - | E4 enhancer mutant. |  |
| pHPV16MANSL-III*ME4 | pHPV16AN | - “ - | E4 enhancer mutant. |  |
| pCMVCAT16 |  | CMV promoter-driven CAT gene with HPV-16 late polyA signal. Lacks late UTR NRE. |  |  |
