# Supplementary material for: Eight Nucleotide Substitutions Inhibit Splicing to HPV-16 3′-Splice Site SA3358 and Reduce the Efficiency by which HPV-16 Increases the Life Span of Primary Human Keratinocytes
Source: PLoS One. 2013 Sep 9;8(9):e72776. doi: 10.1371/journal.pone.0072776 (PMC3767658; doi:10.1371/journal.pone.0072776)
Supplement: Table S2 — List of primers. (DOCX) [file pone.0072776.s003.docx]

**List of primers**

| **Primer name** | **Primer sequences in 5’ to 3’ direction.** |
| --- | --- |
| 757s | CGGTTGTGCGTACAAAGCACACACG |
| E4A | TGCTGCCTAATAGTTTCAGGAGAGG |
| L1A | GCAACATATTCATCCGTGCTTACAACC |
| ExonE2as | CCTGACCACCCGCATGAACTTCC |
| E6S | CCCAGAAAGTTACCACAGTTATGCAC |
| 757as | CGTGTGTGCTTTGTACGCACAACCG |
| 16S | TATGTATGGTATAATAAACACGTGTGTATGTG |
| 16A | GCAGTGCAGGTCAGGAAAACAGGGATTTGGC |
| HPV1e4for | TTTTGGGTACCGCGCGCCTCCCCAAGGGCTGCTGGGGCTCCTGCAG |
| HPV1e4rev | TTTTTTCTAGACTGAGACACTTAAGCTGATTGGCACCC |
| HPV5e4for | TTTTGGGTACCGCGCGCCTCCACGCCTCCAGGGTCGCCAGGAGG |
| HPV5e4rev2 | TTTTTTCTAGAGTGGACCGGGACCTGGTGGTGGACCGAGTG |
| HPV5e4rev1 | TTTTTTCTAGACTTCTTCCTCTGGTTTCGGTTTGTTGTGGCTGT |
| HPV6e4for | TTTTGGGTACCGCGCGCCACTACACAAGAAGTATCCATTCCTGAATCTACTAC |
| HPV6e4rev | TTTTTTCTAGACTTGAAATTGCACTATAGGCGTAGCTGAACTGTTAC |
| HPV18e4for | TTTTGGGTACCGCGCGCTACCAGTGACGACACGGTATCCGCTACTCAGCTTG |
| HPV18e4rev | TTTTTTCTAGACTTTTAAATGTATTATAGGCGTAGTGTTACCACTACAGAGTTTCC |
| HPV41e4for | TTTTGGGTACCGCGCGCCTCCTCCACGAGGGAGAGAACCCCAAA |
| HPV41e4rev | TTTTTTCTAGACTTAAGCACCGCAGGCTATTCACTGGACCTTT |
| 127FT | GGCGTGTGTCTAACACAGACGACTATCCAGCG |
| 128FS | AACACAGACGACTATCCAGCG |
| 129RT | AGACACACGCCCAAGGCGACGGCTTTGGTATGGG |
| 130RS | CAAGGCGACGGCTTTGGTATGGG |
| 135FT | GGCGCCTAAAAAACACAGACGACTATCCAGCG |
| 136RT | TTTTAGGCGCCCAAGGCGACGGCTTTGGTATGGG |
| 137FT | GGCCCCTAAAAAACACAGACGACTATCCAGCG |
| 138RT | TTTTAGGGGCCCAAGGCGACGGCTTTGGTATGGG |
| 139FT | GGCACGGAGGAAACACAGACGACTATCCAGCG |
| 140RT | TCCTCCGTGCCCAAGGCGACGGCTTTGGTATGGG |
| 143FT | AACAGCAACACTACACCCATCGTCCATTTAAAAGGTGATGCTAATACTTTAAAATG |
| 156FT | GGCCCCGAAGAAACACAGACGACTATCCAGCG |
| 157RT | TCTTCGGGGCCCAAGGCGACGGCTTTGGTATGGG |
| 158FT | GGCACCTAAGAAACACAGACGACTATCCAGCG |
| 159RT | TCTTAGGTGCCCAAGGCGACGGCTTTGGTATGGG |
| 160FT | GGCACCGAAAAAACACAGACGACTATCCAGCG |
| 161RT | TTTTCGGTGCCCAAGGCGACGGCTTTGGTATGGG |
| 162FT | GGCTCCGAAGAAACACAGACGACTATCCAGCG |
| 163RT | TCTTCGGAGCCCAAGGCGACGGCTTTGGTATGGG |
| 164RT | TAAATGGACGATGGGTGTAGTGTTGCTGTTACAGTTAATCCGTCCTTTGTGTGAGCTG |
| lucS | ACGCGTGAAATGGAAGACGCCAAAAACATAAAG |
| lucA | CTCGAGTTACACGGCGATCTTTCCGCCCTT |
| slucS | GACGCGTGAAATGGACATCAAGGTGGTGTTC |
| sLucA | CTCGAGTCACCTGTCGCCGGCCATGC |
| gapdhs | AGGTCGGAGTCAACGGATTTGG |
| gapdha | ACAGTCTTCTGGGTGGCAGTGATG |
